# Versatile ferrous oxidation–xylenol orange assay for high-throughput screening of lipoxygenase activity

**DOI:** 10.1007/s00253-024-13095-5

**Published:** 2024-03-18

**Authors:** Ruth Chrisnasari, Tom A. Ewing, Roelant Hilgers, Willem J. H. van Berkel, Jean-Paul Vincken, Marie Hennebelle

**Affiliations:** 1 Laboratory of Food Chemistry, Wageningen University & Research, Bornse Weilanden 9, 6708 WG Wageningen, The Netherlands; 2 Wageningen Food & Biobased Research, Wageningen University & Research, Bornse Weilanden 9, 6708 WG Wageningen, The Netherlands; 3 Faculty of Biotechnology, University of Surabaya (UBAYA), Surabaya, 60293 Indonesia

**Keywords:** Fatty acids, FOX assay, High-throughput screening, Hydroperoxides, Lipoxygenase

## Abstract

**Abstract:**

Lipoxygenases (LOXs) catalyze dioxygenation of polyunsaturated fatty acids (PUFAs) into fatty acid hydroperoxides (FAHPs), which can be further transformed into a number of value-added compounds. LOXs have garnered interest as biocatalysts for various industrial applications. Therefore, a high-throughput LOX activity assay is essential to evaluate their performance under different conditions. This study aimed to enhance the suitability of the ferrous-oxidized xylenol orange (FOX) assay for screening LOX activity across a wide pH range with different PUFAs. The narrow linear detection range of the standard FOX assay restricts its utility in screening LOX activity. To address this, the concentration of perchloric acid in the xylenol orange reagent was adjusted. The modified assay exhibited a fivefold expansion in the linear detection range for hydroperoxides and accommodated samples with pH values ranging from 3 to 10. The assay could quantify various hydroperoxide species, indicating its applicability in assessing LOX substrate preferences. Due to sensitivity to pH, buffer types, and hydroperoxide species, the assay required calibration using the respective standard compound diluted in the same buffer as the measured sample. The use of correction factors is suggested when financial constraints limit the use of FAHP standard compounds in routine LOX substrate preference analysis. FAHP quantification by the modified FOX assay aligned well with results obtained using the commonly used conjugated diene method, while offering a quicker and broader sample pH range assessment. Thus, the modified FOX assay can be used as a reliable high-throughput screening method for determining LOX activity.

**Key points:**

• *Modifying perchloric acid level in FOX reagent expands its linear detection range*

• *The modified FOX assay is applicable for screening LOX activity in a wide pH range*

• *The modified FOX assay effectively assesses substrate specificity of LOX*

**Supplementary Information:**

The online version contains supplementary material available at 10.1007/s00253-024-13095-5.

## Introduction

**Fig. 1:**
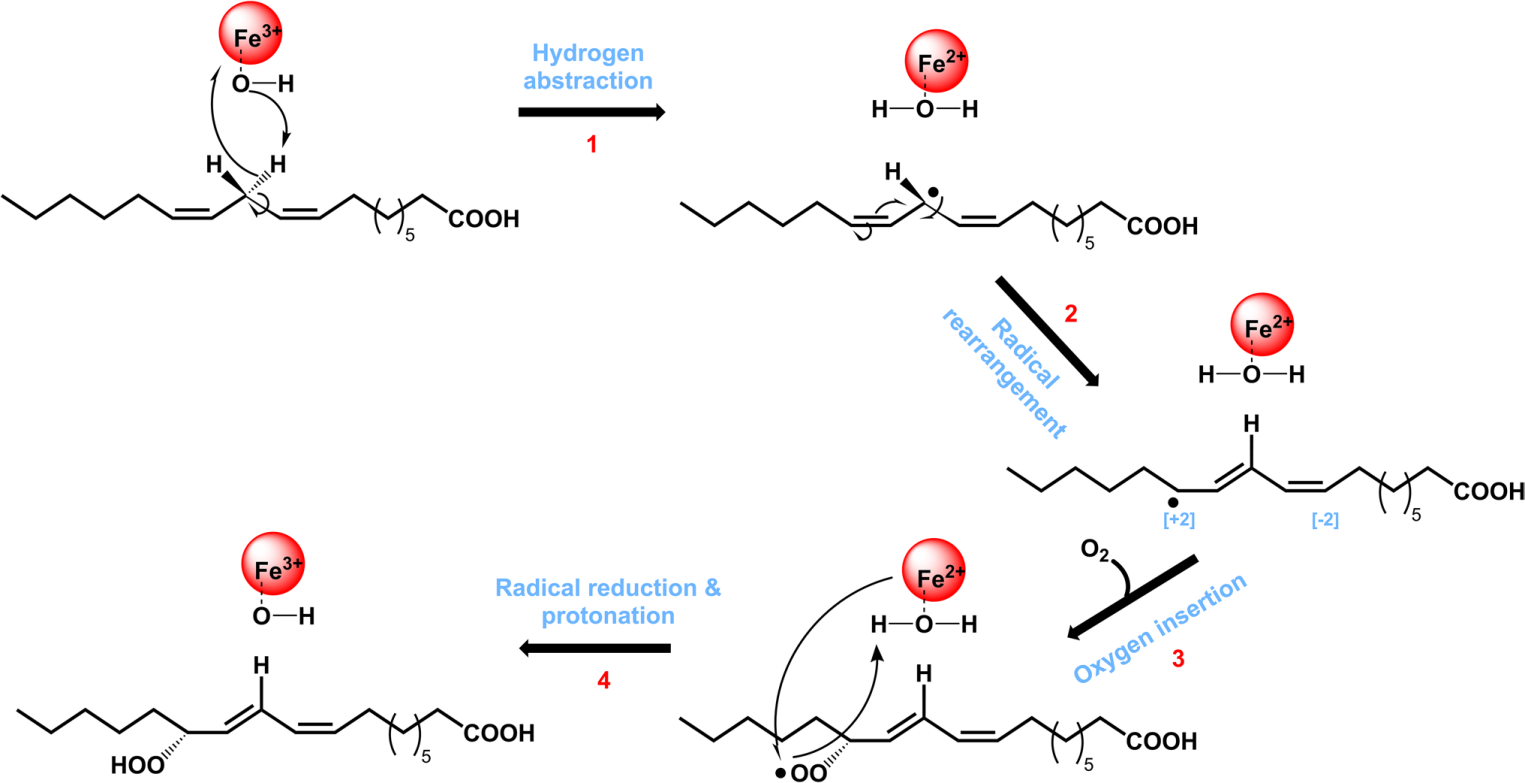
Reaction mechanism of lipoxygenase (LOX). LOX catalyzes the oxygenation of fatty acids through four reaction steps: hydrogen abstraction, radical rearrangement, oxygen insertion, and radical reduction-protonation. The figure is adopted from Chrisnasari et al. (2022) with slight modification

Lipoxygenases (LOXs; EC 1.13.11.x) are non-heme iron- (or in some cases manganese-) dependent enzymes that catalyze dioxygenation of polyunsaturated fatty acids (PUFAs) containing a (1*Z*,4*Z*)-pentadiene structural unit, leading to the formation of fatty acid hydroperoxides (FAHPs) containing a (1*Z*,3*E*)-conjugated diene pattern. LOX catalyzes the dioxygenation of fatty acids in four distinct steps (Fig. 1): (1) Ferric iron (Fe^3+^) cofactor initiates the reaction by abstracting a hydrogen atom at the center of the pentadiene structure of the substrate (Lehnert and Solomon 2003), the unpaired electron is transferred to the ferric iron thereby reducing it to the ferrous form (Fe^2+^), and the proton is transferred to the hydroxide ligand that is coordinated to the iron, yielding ferrous iron, water, and a lipid alkyl radical. (2) The unpaired electron generated in the substrate undergoes rearrangement to either the [+ 2] or the [− 2] position relative to the abstracted hydrogen. (3) A dioxygen molecule is introduced leading to the formation of a fatty acid peroxyl radical (ROO•). (4) The fatty acid peroxyl radical is then reduced by an electron from the ferrous iron (Fe^2+^) and protonated, resulting in the formation of FAHP (Hamberg and Samuelsson 1967; Egmond et al. 1972; Hamberg et al. 1998; Lehnert and Solomon 2003). FAHPs produced by LOXs can be transformed to numerous value-added compounds, e.g., hydroxy fatty acids, aldehydes, and alcohols (Gigot et al. 2010; Song et al. 2013). These compounds are of interest to the chemical and food industries because they can be used as precursors for the production of biopolymers, surfactants, and flavor compounds (Gigot et al. 2010; Mutlu and Meier 2010; Liu et al. 2012; Song et al. 2013; Hu et al. 2014).

The industrial potential of LOX has led to the exploration of this enzyme from various sources. Among these sources, microbial LOXs have gained increasing attention in recent years for their ability to act on a wide range of PUFAs (Banthiya et al. 2016; Newie et al. 2016; An et al. 2018; Goloshchapova et al. 2018; Qi et al. 2020; Chrisnasari et al. 2022; Kim et al. 2022) and some of them were optimally active at extreme pH values (Qian et al. 2017; Goloshchapova et al. 2018). These advantages stimulate their exploitation as an industrial biocatalyst in a wide range of applications. To facilitate the biocatalytic exploitation of microbial LOXs, it is essential to establish a robust and reliable high-throughput screening method for LOX activity. Due to the wide range of pH values in which microbial LOXs have shown to be active and their ability to convert a range of different PUFAs, this method should enable the rapid assessment of their activity toward various PUFA substrates at different pH values.

LOX activity can be measured either directly or indirectly (Table 1). Direct methods involve measuring either the formation of LOX products (i.e., FAHPs) or the decrease of substrates or co-substrates (i.e., fatty acids or O_2_), whereas indirect methods monitor the reaction between LOX products and other compounds. Direct measurement of LOX activity can be done by following the formation of FAHPs using spectrophotometric analysis. For example, a commonly used method is to measure the formation of conjugated diene moieties at a wavelength of 233–235 nm (Corongiu and Milia 1983). However, this method is limited in terms of buffer compatibility, as many chemicals used for buffer preparation (e.g., citric acid (Krukowski et al. 2017), glycine (Gao and Zhang 2018), potassium hydrogen phthalate (Kim et al. 2016), acetic acid (Šuňovská et al. 2012), Bis–Tris (based on our observation)) can interfere with absorbance measurements at this wavelength thus limiting the applicability of the method for pH optimum screening. Moreover, different FAHPs have varying extinction coefficients (Browne and Armstrong 1998), which makes direct comparison of LOX activity toward different PUFAs challenging, unless all respective standards are available. Another common direct method for determining LOX activity is by measuring oxygen consumption using an oxygen electrode. This method allows for LOX activity to be measured independently of the buffer used (Berkeley and Galliard 1976). However, the disadvantage of this method is its low throughput, limiting its application for rapid screening of many samples.

**Table 1:** Reported methods to measure LOX activity

Method	Working pH range	Linear detection range	Interference	Other limitations
Direct methods:				
Conjugated diene	Depends on the buffer selection	0–40 µM (Pinto et al. 2007)	Chemicals used for buffer preparation, e.g., citric acid, acetic acid, Bis–Tris	Different response of different FAHPs (Browne and Armstrong 1998)
O_2_ consumption	Independent of pH	n.r	n.r	Low throughput
Indirect methods:				
Bleaching assay	n.r	n.r	Pigments (Romero and Barrett 1997)	–
Fluorescein	pH > 6.4 (Le Guern et al. 2020)	n.r	Antioxidants, reducing agents, and radical scavengers (Whent et al. 2010)	–
MBTH-DMAB	n.r	0–35 µM (Anthon and Barrett 2001)	n.r	Sensitive to the changes in the Hb-LOX ratio (Anthon and Barrett 2001), Hb has a quasi-LOX activity (Kuhn et al. 1981)
FOX assay	n.r	0–25 µM (Pinto et al. 2007)	n.r	Low linear detection ranges (Nielsen et al. 2003; Pinto et al. 2007), different response of different hydroperoxides (Jiang et al. 1992; Gay et al. 1999a; Vega et al. 2005)

*n.r.* not reported

In addition to these direct methods, some indirect methods have been developed for the rapid measurement of LOX activity. Bleaching assays are used to assess the ability of LOX enzymes to bleach dyes (Romero and Barrett 1997; Qian et al. 2017; Lu et al. 2020). In these assays, LOX oxidizes fatty acids to FAHPs, which subsequently oxidize the dye. However, the sensitivity of bleaching assays is affected by the presence of impurities in the enzyme solution, i.e., pigments (Romero and Barrett 1997), therefore limiting its application for screening the activity of crude LOXs sourced from plants. Another indirect method using fluorescein has shown excellent reproducibility, accuracy, and precision (Whent et al. 2010). Fluorescein has been proposed to act as a scavenger by reacting with the peroxyl radical (ROO•) formed during reactions catalyzed by LOX, which can be monitored by looking at the decrease in fluorescein fluorescence (Whent et al. 2010). Nevertheless, fluorescein can only be used in a pH range above its p*K*a value of 6.4. At lower pH values, the protonated forms of fluorescein become non-fluorescent (Le Guern et al. 2020), thus limiting the use of this method for pH optimum screening. In another indirect method, FAHPs produced by LOX act as oxidants, while hemoglobin (Hb) acts as a catalyst for oxidative coupling of 3-methyl-2-benzothiazolinone hydrazone (MBTH) with 3-(dimethylamino)benzoic acid (Anthon and Barrett 2001). The resulting reaction produces a purple color that can be detected spectrophotometrically. Even though the method was shown to give comparable results to the conjugated diene method, the sensitivity to changes in the Hb-LOX ratio makes this method hard to apply, especially when an unknown concentration of LOX or impure LOX is used (Anthon and Barrett 2001). Moreover, Hb has been reported to have quasi-LOX activity when a high concentration of substrate is used (i.e., more than 0.1 mM linoleic acid) and the reaction is measured at a pH between 7 and 9 (Kuhn et al. 1981), increasing the complexity of the method and making it unsuitable for optimum pH screening of LOXs.

Among the indirect methods, the ferrous oxidation–xylenol orange (FOX) assay has been reported as a sensitive method applicable for high-throughput screening of LOX activity (Waslidge and Hayes 1995; Cho et al. 2006; Li and Schwarz 2018), which can detect up to 1.0–2.5 µM of FAHPs (Cho et al. 2006; Pinto et al. 2007). The assay is a widely used method to determine the hydroperoxide content of biological and food samples (Bou et al. 2008), as well as to assess the activity of LOX extracted from plant and animal material (Vega et al. 2005; Pinto et al. 2007; Fukuzawa et al. 2009; Timabud et al. 2013). The assay is based on the oxidation of ferrous iron (Fe^2+^) to ferric iron (Fe^3+^) by FAHPs produced by LOX. The resulting ferric iron subsequently forms a complex with xylenol orange (Fe^3+^–XO complex) which gives a visible purple color and absorbs strongly at 550–580 nm (Bou et al. 2008). To obtain accurate results, it is crucial to conduct the FOX assay under acidic conditions. This is necessary because ferrous ions tend to rapidly convert to ferric ions in non-acidic environments (Straub et al. 2001). By maintaining the reaction mixture under acidic conditions, the oxidation of ferrous ions occurs specifically due to the presence of hydroperoxides (Wolff 1994). This ensures that only the desired reaction takes place, leading to reliable and accurate results in the assay. The effect of different acids on the sensitivity of the assay has been evaluated (Gay et al. 1999a; Gay and Gebicki 2002; Vega et al. 2005). Perchloric acid was proposed to be more suitable than sulfuric acid because it decreases the sensitivity to pH shifts and increases the ability to tolerate the presence of biological materials (Gay and Gebicki 2002). When buffers with varying pH values are used for pH optimum screening of LOX activity, the final pH of the reaction mixture might be slightly altered and thereby the sensitivity of the assay may be affected. However, the applicability of this method across a wide range of sample pH has not been evaluated yet.

A current limitation of the FOX assay is its low linear detection range (Nielsen et al. 2003; Pinto et al. 2007), which is unfavorable for accurately measuring LOX-produced FAHPs, especially when their concentration is expected to be high. At such elevated levels, accurate measurements become impossible since they exceed the detection limit of the assay. In addition, attempting to stop the enzymatic reaction and dilute the sample afterward is not a feasible solution due to the instability of FAHPs (Griffiths et al. 2000; Musakhanian et al. 2022). As a result of this restricted linear detection range, adjustments to the enzymatic reaction mixture become necessary, such as modifying the enzyme and/or substrate concentrations and/or incubation time, to facilitate the quantification of the generated FAHPs. Adjustments of those abovementioned conditions take effort and time, especially when the activity of the LOX of interest is unknown, which affects the simplicity of the assay. Therefore, it is important to find a way to extend the linear detection range. Another potential limitation of the FOX assay is that different hydroperoxide species can exhibit different reactivity toward the xylenol orange reagent (Jiang et al. 1992; Gay et al. 1999a; Vega et al. 2005), causing difficulties for the accurate quantification of multiple different LOX-produced FAHPs. So far, cumene hydroperoxide (CuHP) is the most commonly used standard compound for calibration in the FOX assay. However, it shows a different response compared to the tested FAHPs, i.e., the response of linoleic acid hydroperoxide is 54% higher than that of CuHP, while those of linolenic and arachidonic acid hydroperoxide are 18% and 27% lower than that of CuHP, respectively (Vega et al. 2005). Evaluation of the response of other FAHP species has not been reported yet.

In this study, we report a modification of the FOX assay. By increasing the concentration of perchloric acid used in the FOX reagent, we aim at extending the linear detection range of hydroperoxides. This is because at very acidic conditions, ferrous iron becomes less prone to oxidation and xylenol orange becomes fully protonated (Liosi et al. 2017). This slows down the complexation reaction and reduces the sensitivity of the assay. In order to maintain a comparable level of detection, higher concentrations of hydroperoxides are required, thereby extending the linear detection range. Optimization of the perchloric acid concentration is necessary to strike a balance between expanding the linear detection range of hydroperoxides while maintaining sufficient sensitivity. In this study, we tested four different perchloric acid concentrations to evaluate the sensitivity and linear detection range of the assay. We also assessed the capability of the modified FOX assay to measure hydroperoxides in samples with a wide range of pH values. Furthermore, we evaluated the assay’s ability to measure different hydroperoxide species, which is of importance for its applicability for screening the substrate specificity of LOXs. Finally, we compared the performance of the modified FOX assay in determining FAHP produced by a bacterial LOX to the commonly used conjugated diene method.

## Materials and methods

### Materials

Chemicals used for enzymatic reactions and FOX assay were obtained from the following sources: linoleic acid (LA; C18:2 Δ9*Z*,12*Z*) from Nu-Chek Prep, Inc., Minnesota, USA; fatty acid hydroperoxide standards (13(*S*)-hydroperoxy-9*Z*,11*E*-octadecadienoic acid (13-HPODE), 13(*S*)-hydroperoxy-9*Z*,11*E*,15*Z*-octadecatrienoic acid (13-HPOTE), 15(*S*)-hydroperoxy-5*Z*,8*Z*,11Z,13*E*-eicosatetraenoic acid (15-HPETE), 12(*S*)-hydroperoxy-5*Z*,8*Z*,10*E*,14*Z*,17*Z*-eicosapentaenoic acid (12-HPEPE), and 17(*S*)-hydroperoxy-4*Z*,7*Z*,10*Z*,13*Z*,15*E*,19*Z*-docosahexaenoic acid (17-HPDHE)) from Larodan, Solna, Sweden; xylenol orange tetrasodium salt, iron(II) sulfate heptahydrate, cumene hydroperoxide (CuHP), hydrogen peroxide (HP), and perchloric acid from Sigma-Aldrich, Missouri, USA.

The gene of *Burkholderia thailandensis* lipoxygenase (Bt-LOX) (An et al. 2015) (NCBI ABC36974.1) with codons optimized for expression in *Escherichia coli* (Table S1) was purchased from GenScript Biotech, The Netherlands. The gene was obtained in a pET-19b plasmid (Novagen, USA), inserted between the *Nde*I and *Blp*I restriction sites. A 10 × His-tag was present at the N terminus of the enzyme and was used for protein purification. Materials used for the enzyme production and purification were obtained from the following sources: *Escherichia coli* BL21(DE3) competent cells from Invitrogen, California, USA; Luria Bertani medium, pepstatin A, and ampicillin sodium salt from Sigma-Aldrich, Missouri, USA; isopropyl β-d-1-thiogalactopyranoside (IPTG) from Duchefa Biochemie B.V., Haarlem, The Netherlands; BugBuster master mix and Ni–NTA His-bind resin from Millipore-Merck, Darmstadt, Germany; cOmplete mini EDTA-free protease inhibitor cocktail from Roche, Mannheim, Germany; VivaSpin spin filters from GE Healthcare, Buckinghamshire, UK.

### Protein expression and purification

Recombinant *E. coli* BL21(DE3) transformed with the pET-19b_Bt-LOX plasmid was cultivated in Luria Bertani medium at 37 °C with shaking at 250 rpm. When the optical density of the bacterial culture at 600 nm (OD_600_) reached 0.6 − 0.8, 0.5 mM IPTG was added and the culture was further incubated at 16 °C with shaking at 150 rpm for 48 h. Then, the cells were harvested by centrifugation at 7000 × *g* for 15 min at 4 °C and stored at − 20 °C until protein purification.

To purify the Bt-LOX enzyme, a lysis solution was first added to the frozen cell pellet obtained from 200 mL culture medium. The lysis solution consisted of one Mini EDTA-free cOmplete protease inhibitor cocktail tablet and 1 µM pepstatin A dissolved in 10 mL of BugBuster Master Mix. Centrifugation at 16,000 × *g* for 20 min at 4 °C was carried out to remove cell debris, and the resulting supernatant was filtered using a 0.22-μm membrane filter. Subsequently, purification was conducted using a gravity flow column containing 1 mL of Ni–NTA His-bind resin. Prior to sample application, the column was equilibrated with 10 column volumes (CV) of an equilibration buffer consisting of 50 mM NaH_2_PO_4_, 300 mM NaCl, and 10 mM imidazole adjusted to pH 7.0 with a 1 M HCl solution. The filtered supernatant was then applied to the column, which was subsequently washed with 2 CV each of four washing buffers pH 7, containing 50 mM NaH_2_PO_4_, 300 mM NaCl, and increasing concentrations of imidazole of 20, 50, 100, and 150 mM, respectively. Elution of the purified enzyme was achieved using 4 CV of elution buffer pH 7, containing 50 mM NaH_2_PO_4_, 300 mM NaCl, and 250 mM imidazole. The desalting of the elution fractions was performed using a VivaSpin spin filter with a molecular weight cut-off of 10 kDa. The purified enzyme was stored in 100 mM phosphate buffer pH 7, its concentration was determined using the BCA assay, and its size was confirmed by SDS-PAGE, showing a single band at approximately 75 kDa.

### Absorption spectral analysis of the ferric-xylenol orange complex

The FOX assay was carried out with some modifications of the previously described method (Gay and Gebicki 2002; Pinto et al. 2007). The xylenol orange reagent containing 2.0 mM ferrous sulfate, 0.29 mM xylenol orange tetrasodium salt, and 440 mM perchloric acid in methanol/water (9:1) was freshly prepared. To find the optimum absorbance at which the concentration of the ferric-xylenol orange (Fe^3+^–XO) complex can be determined, 30 µL of cumene hydroperoxide (0–10.52 mM dissolved in water) and 150 µL xylenol orange reagent were mixed well. The mixtures were then incubated for 15 min at room temperature (~ 20 °C) and the absorption spectra of the mixtures were measured in the wavelength range of 400–650 nm using a Spectramax ID3 multi-detection microplate reader (Molecular Devices, LLC, San Jose, California, USA). When the absorbance of the samples exceeded 2.0, the samples were diluted in 75% methanol in water. The actual absorbance of the sample was then calculated, taking the dilution factor into account.

### Optimization of perchloric acid concentration and its effect on the linear detection range of hydroperoxides

To study the effect of perchloric acid concentration on the linear detection range of the FOX assay, xylenol orange reagent was freshly prepared using different concentrations of perchloric acid. The xylenol orange reagent contained 2.0 mM ferrous sulfate, 0.29 mM xylenol orange tetrasodium salt, and perchloric acid (110, 220, 440, or 660 mM) diluted in methanol/water (9:1). The assay was carried out by mixing 30 μL of the sample and 150 μL xylenol orange reagent in a 96-well microplate. The mixture was then incubated for 15 min at room temperature. The absorbance of the samples was read at 570 nm using the Spectramax ID3 multi detection microplate reader. The concentration of perchloric acid which offers a broader linear detection range while maintaining sufficient sensitivity for the measurement (440 mM) was then selected for further experiments.

### Evaluation of the modified FOX assay on different sample pHs and different hydroperoxide species

The effect of the sample pH on the modified FOX assay was evaluated by performing the assay on CuHP calibration curves (0–175.2 µM) diluted in different buffers. The buffers used were 100 mM citrate pH 3.0, 4.0, and 5.0, 100 mM Bis–Tris pH 6.0 and 7.0, and 100 mM Tris–HCl pH 8.0, 9.0, and 10.0. The effect of different hydroperoxide species on the response of the modified FOX assay was assessed by measuring various concentrations of the hydroperoxide species, i.e., CuHP, HP, 13-HPODE, 13-HPOTE, 15-HPETE, 12-HPEPE, and 17-HPDHE. For the sake of stability, all the hydroperoxide standard compounds were dissolved in methanol. When measuring different hydroperoxide species, the methanol/water ratio in the xylenol orange reagent was adjusted to 7:1. This adjustment ensured that the final methanol concentration in the reaction mixture remained consistent with the standard protocol. The molar absorption coefficient (Ɛ) of the Fe^3+^–XO complex for each hydroperoxide was calculated using Eq. (1). The absorbance of the Fe^3+^–XO complex at 570 nm (*A*) per concentration of the hydroperoxide (*c*) was determined from the slope of the linear part (*R*^2^ > 0.99) of the calibration curve. The length of the light path through the solution (*l*) was determined by calculating the height of the sample solution in the 96-well microplate.

1 $$\varepsilon =\frac{A}{l c}$$

### Determination of Fe^3+^/hydroperoxide ratio

The number of ferric ions generated by each –OOH group from different hydroperoxide species was expressed as the Fe^3+^/hydroperoxide ratio. The calculation involved dividing the molar absorption coefficient of the Fe^3+^–XO complex generated by the –OOH group in each hydroperoxide species by the molar absorption coefficient of the complex generated by ferric ions in the same reagent (Gay et al. 1999b, 1999a). The molar absorption coefficient of the Fe^3+^–XO complex generated by ferric ions was determined by making a calibration curve of FeCl_3_ from 0 to 120 µM.

### Preparation of solubilized fatty acids

Solubilized LA was freshly prepared according to a previously described protocol with slight modifications (Axelrod et al. 1981). In a 10-mL volumetric flask, 13.5 μL LA was mixed with 12.5 μL of Tween-20 in 4 mL milli-Q water. After adding 0.55 mL of 0.5 M NaOH, the solution became clear, and milli-Q water was added to adjust the volume to 10 mL, resulting in a final LA concentration of 4.3 mM.

### Comparison between FOX assay and conjugated diene method

To validate the accuracy of the modified FOX assay, fatty acid hydroperoxide produced from LA by Bt-LOX was measured using two different methods, i.e., FOX assay and conjugated diene method. Solutions containing 13.25 nM Bt-LOX, LA in the range of 4.3–139 µM, and 100 mM buffer pH 6 were used as the enzymatic reaction mixtures. Bis–Tris buffer was used for the FOX assay, while phosphate buffer was used for the conjugated diene method to prevent any interference from absorption by the buffer. The reaction mixtures were homogenized and incubated for 5 min at 25 °C. LOX activity measured based on the FOX assay was performed as described above; 13-HPODE (LA-derived hydroperoxide) was used as the standard for the calibration curve. Concentration of fatty acid hydroperoxide based on the conjugated diene method was determined by measuring the absorbance at 234 nm during 5-min incubation of the enzyme with LA using Jasco V-730 UV–vis/NIR spectrophotometer (Jasco, Easton, Maryland, USA). The concentration of fatty acid hydroperoxide formed was calculated using a molar absorption coefficient of 25,000 M^−1^ cm^−1^ (Axelrod et al. 1981).

## Results

### Absorption spectral analysis of the Fe^3+^–XO complex

**Fig. 2:**
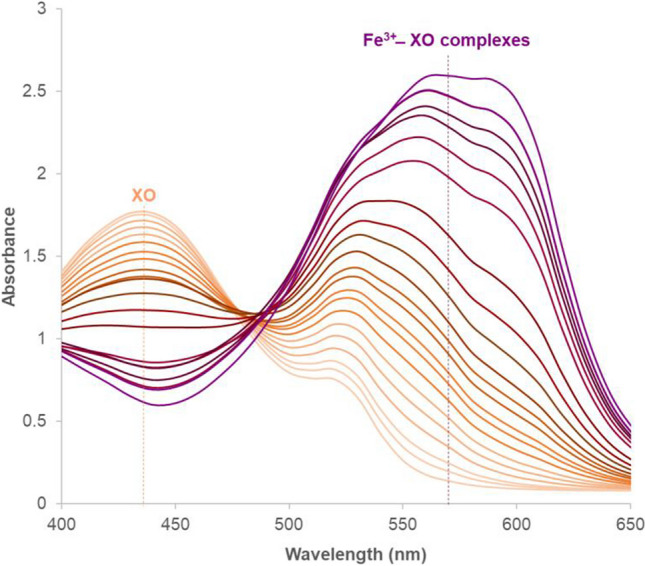
Absorption spectra of xylenol orange reagent in the presence of cumene hydroperoxide at 0, 8.8, 17.5, 35.0, 52.6, 70.1, 87.6, 105.1, 122.7, 140.2, 157.7, 227.8, 262.8, 297.9, 368.0, 438.0, 525.7, 613.3, 788.5, 1226.5, and 10520.0 µM (shown as lines colored progressively from light orange to dark purple). Xylenol orange shows the highest peak centered at 440 nm (shown as lightest orange line) that decreases in the presence of cumene hydroperoxide due to the formation of Fe^3+^ − XO complexes, characterized by a progressive shift in the maximum absorbance, starting from 520 to 530 nm, then to 560 nm, and finally to 590 nm. Samples with absorbance values surpassing 2.0 were diluted in a 75% methanol–water solution, and the final absorbance was determined considering the dilution factor

In the FOX assay, ferrous iron is oxidized to ferric iron in the presence of hydroperoxides, which can then react with xylenol orange to form the Fe^3+^ − XO complex. To determine at which wavelength the Fe^3+^ − XO complex shows the highest absorbance and should therefore be measured in the assay, 0 to 10.52 mM of CuHP was incubated in the presence of xylenol orange reagent and the absorbance of the mixture was measured in the wavelength range of 400–650 nm. In the absence of CuHP, the absorption spectrum of XO exhibited two distinct peaks at 440 and 520 nm (Fig. 2), which agrees with previous reports (Timabud et al. 2013; Belleza and Villaraza 2014). The peak observed at 440 nm (dashed orange line), which indicates the presence of xylenol orange, has been frequently reported (Jiang et al. 1992; Hermes-Lima et al. 1995; Gay et al. 1999a; Timabud et al. 2013). On the other hand, the peak at 520 nm may indicate the formation of a complex between Fe^2+^ and XO (Fig. S1), as xylenol orange has also been reported to form complexes with divalent metal ions (Belleza and Villaraza 2014). The gradual increase in CuHP concentration led to a progressive shift in the maximum absorbance, starting from 520 nm and progressing to 530 nm, then to 560 nm, and finally to 590 nm (Fig. 2). The spectral shift toward higher wavelengths occurred due to the presence of CuHP, which oxidized ferrous iron to its ferric state leading to the formation of Fe^3+^ − XO complexes. The shift in the maximum absorbance to four distinct wavelengths suggests the existence of different Fe^3+^ − XO complexes at potentially four different stoichiometric ratios: (Fe^3+^)–(XO)_3_, (Fe^3+^)–(XO)_2_, (Fe^3+^)–(XO), and (Fe^3+^)_2_–(XO) (Yoshino et al. 1979; Liosi et al. 2017; Scotti et al. 2022). The formation of different Fe^3+^ − XO complexes is also indicated by the absence of an isosbestic point. Due to interference caused by the initial state of the xylenol orange reagent at 520 nm, the wavelength of 570 nm (indicated by the dashed purple line), which is near the midpoint between 560 and 590 nm, was chosen for further analysis. From this point onwards, the term “Fe^3+^ − XO complex” will be used to indicate complexes formed at any stoichiometric ratio of Fe^3+^ and XO, without specifying which of the possible complexes are formed.

### Effect of perchloric acid concentration on the linear detection range of hydroperoxides

**Fig. 3:**
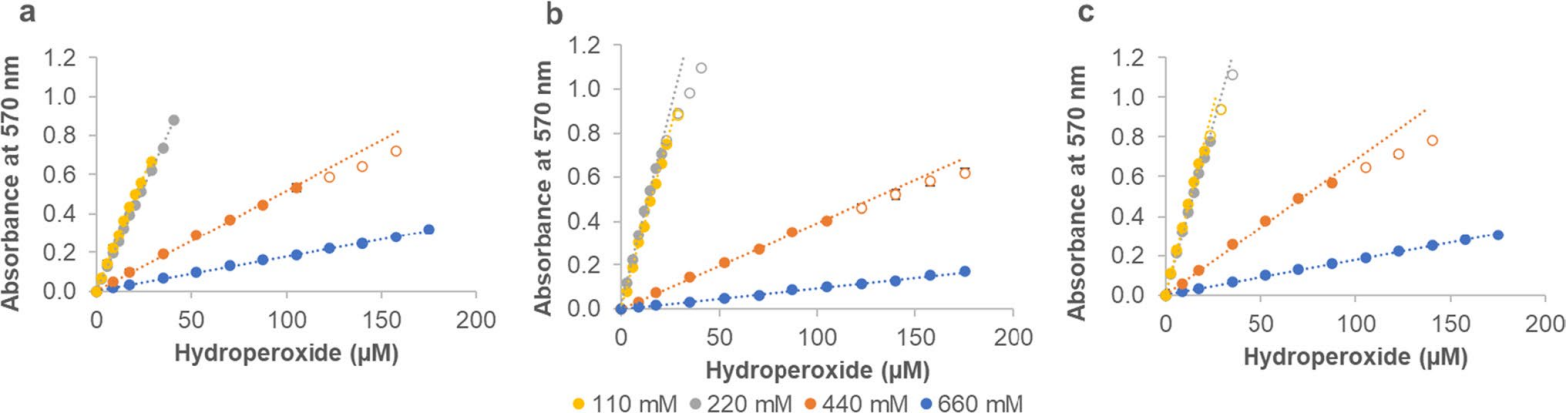
Effect of perchloric acid concentration (110, 220, 440, and 660 mM) in the xylenol orange reagent on the linear detection range of cumene hydroperoxide diluted in different buffers. **a** 100 mM citrate buffer pH 3.0, **b** 100 mM Bis–Tris pH 6.0, and **c** 100 mM Tris–HCl pH 9.0. The linear ranges (*R*^2^ value > 0.99) are represented by filled circles, while datapoints outside the linear ranges are indicated by open circles of the same color. Data represent mean ± SD (*n* = 3). When not visible, the error bars are hidden below the markers

The effect of perchloric acid concentration in the FOX reagent on expanding the linear detection range of hydroperoxides was evaluated. As hydroperoxide production by LOXs is commonly conducted in a buffered system, calibration curves of CuHP dissolved in various buffers were measured using xylenol orange reagent dissolved in different concentrations of perchloric acid (110, 220, 440, and 660 mM). The results showed that increasing the concentration of perchloric acid expands the linear detection range of CuHP in all the buffers and pH values tested. Figure 3a, b, and c illustrate the results for 100 mM citrate buffer pH 3.0, 100 mM Bis–Tris pH 6.0, and 100 mM Tris–HCl pH 9.0, respectively (the complete data set at all different pH values is shown in Fig. S2). Concentrations of 110 and 220 mM perchloric acid gave a comparable linear range for CuHP from 0 to 20–35 µM, depending on the pH and the type of buffer employed. This finding is similar to the previous report that xylenol orange with 110 mM perchloric acid gives a linear range for hydroperoxy linoleic acid from 0 to 25 µM (Pinto et al. 2007). Higher concentrations of perchloric acid of 440 and 660 mM gave broader linear ranges for CuHP from 0 to 88–105 µM and from 0 to 175 µM, respectively. However, the sensitivity of the method is diminished by the increased perchloric acid concentrations, as the molar absorption coefficient of the Fe^3+^–XO complex decreases (Table 2). Depending on the pH and the type of buffer employed, the molar absorption coefficient of the Fe^3+^–XO complex decreases up to five–ninefold at 440 mM perchloric acid and up to 15–34-fold at 660 mM perchloric acid compared to that in 110 mM perchloric acid. The gradual decrease in the sensitivity of detection with increasing concentration of perchloric acid has been previously reported for concentrations between 110 and 180 mM (Gay and Gebicki 2002). Altogether, 440 mM perchloric acid provided a wider linear range compared to lower concentrations, while maintaining sufficient sensitivity for the measurement (i.e., with a higher molar absorption coefficient compared to 660 mM perchloric acid). The very low measured absorbance at 570 nm under 660 mM perchloric acids concentration (less than 0.1 for some data points as presented in Fig. S3d) makes the measurement less reliable. For this reason, 440 mM perchloric acid concentration was used in the following experiments.

**Table 2:** Molar absorption coefficients of the Fe^3+^–XO complex for cumene hydroperoxide in presence of various buffer types at different pH values measured by FOX assay using various concentrations of perchloric acid

Buffer	Molar absorption coefficient of Fe^3+^–XO complex at 570 nm (M^−1^ cm^−1^)			
	110 mM^*^	220 mM^*^	440 mM^*^	660 mM^*^
Citrate pH 3	51,068 ± 514	46,460 ± 620	10,597 ± 243	3,726 ± 68
Citrate pH 4	63,774 ± 1091	61,131 ± 509	11,456 ± 169	3,828 ± 66
Citrate pH 5	62,708 ± 1076	61,553 ± 1206	13,033 ± 225	4,029 ± 44
Bis–Tris pH 6	68,258 ± 3101	64,479 ± 503	8,185 ± 102	2,011 ± 67
Bis–Tris pH 7	88,067 ± 922	70,427 ± 658	9,533 ± 63	3,047 ± 53
Tris–HCl pH 8	61,173 ± 563	56,527 ± 382	8,529 ± 115	2,539 ± 12
Tris–HCl pH 9	77,584 ± 1654	70,907 ± 1160	14,074 ± 65	3,688 ± 20
Tris–HCl pH 10	92,930 ± 1455	73,804 ± 678	17,147 ± 219	4,915 ± 91

^*^Concentration of perchloric acid used in the xylenol orange reagent. Data represent mean ± SD (*n* = 3)

### Effect of buffer type and pH on the molar absorption coefficient of the Fe^3+^ − XO complex

**Fig. 4:**
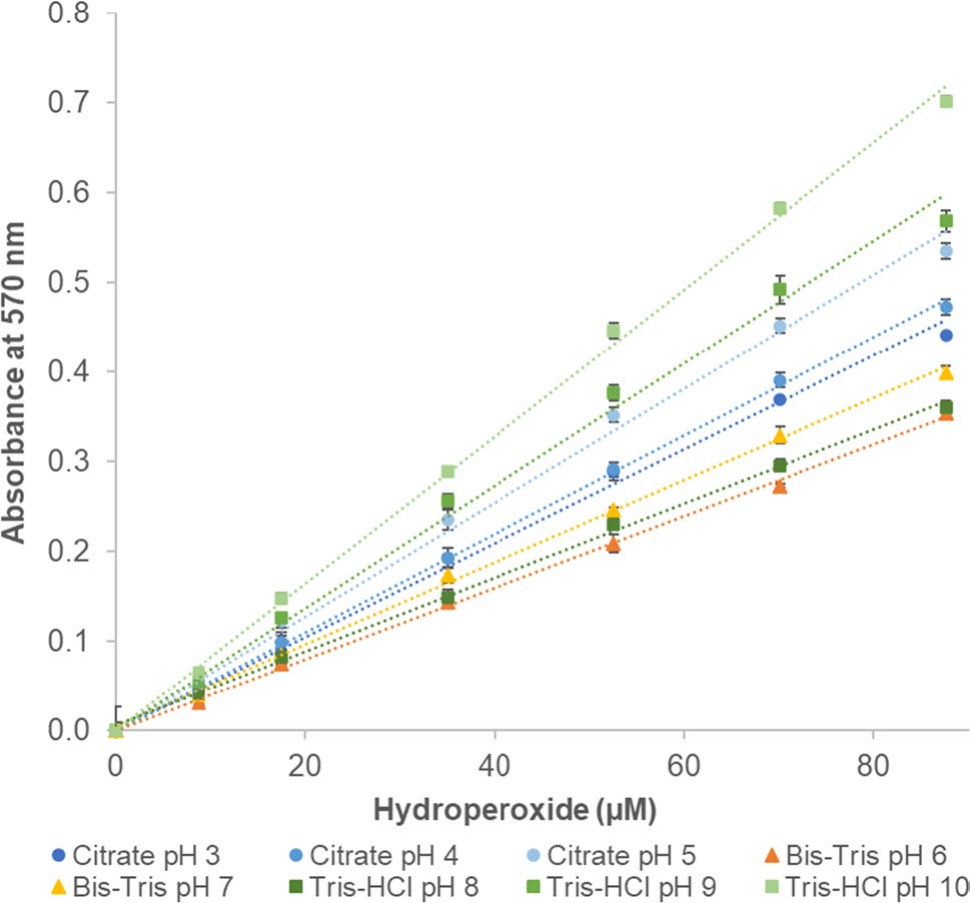
Standard curves of cumene hydroperoxide diluted in different buffers measured by FOX assay. All the buffers used had a concentration of 100 mM. FOX reagent was prepared using 440 mM perchloric acid. Data represent mean ± SD (*n* = 3). When not visible, the error bars are hidden below the markers

To test the applicability of the modified FOX assay to screen for the pH optimum of LOXs, the method was applied to CuHP standard samples prepared in different buffers covering a wide pH range (Fig. S3). A linear range was observed for CuHP at all tested sample pH values (pH 3–10), suggesting that the FOX assay is suitable for measuring the formation of hydroperoxides over a broad range of sample pH (Fig. 4). However, the sensitivity of the assay depends on the type of sample buffer used and its pH. Different types of buffer yielded different molar absorption coefficients for the Fe^3+^ − XO complex. In addition, lowering the sample pH within the same buffer type resulted in a decrease in the molar absorption coefficient of the Fe^3+^ − XO complex (Table 2).

### Sensitivity of the modified FOX assay toward different hydroperoxides species

**Fig. 5:**
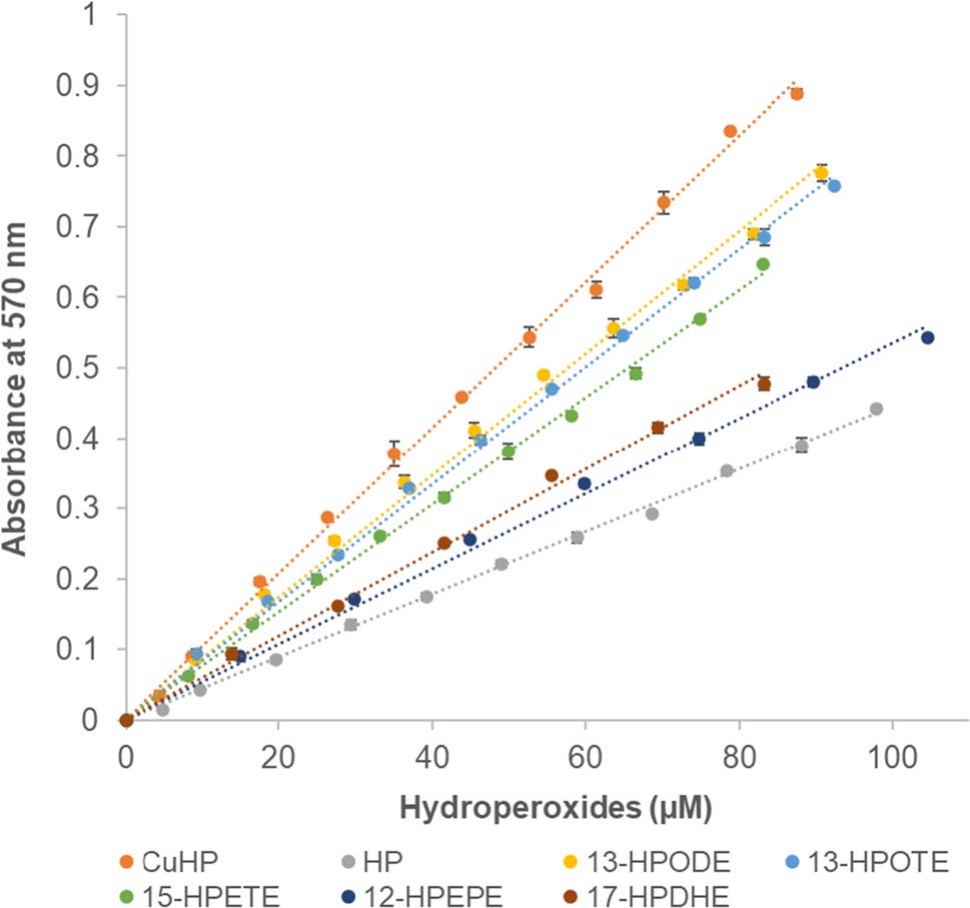
Standard curves of different hydroperoxides measured by the FOX assay using 440 mM of perchloric acid. CuHP: cumene hydroperoxide, HP: hydrogen peroxide, 13-HPODE: 13(*S*)-hydroperoxy-9*Z*,11*E*-octadecadienoic acid, 13-HPOTE: 13(*S*)-hydroperoxy-9*Z*,11*E*,15*Z*-octadecatrienoic acid, 15-HPETE: 15(*S*)-hydroperoxy-5*Z*,8*Z*,11*Z*,13*E*-eicosatetraenoic acid, 12-HPEPE: 12(*S*)-hydroperoxy-5*Z*,8*Z*,10*E*,14*Z*,17*Z*-eicosapentaenoic acid, and 17-HPDHE: 17(*S*)-hydroperoxy-4*Z*,7*Z*,10*Z*,13*Z*,15*E*,19*Z*-docosahexaenoic acid. Error bars represent mean ± SD (*n* = 3). When not visible, the error bars are hidden below the markers

To investigate the sensitivity of the modified FOX assay toward different hydroperoxide species, we measured various concentrations of FAHPs, CuHP, and HP using this assay. The results showed that different hydroperoxide species exhibit different reactivity in the modified FOX assay (Fig. 5), which is indicated by different molar absorption coefficients between the hydroperoxide species (Table 3). To understand this phenomenon, the amount of ferric ions formed per hydroperoxide molecule was estimated for each species. To achieve this, the molar absorption coefficients of Fe^3+^–XO complexes generated by each hydroperoxide species were compared to the molar absorption coefficients of Fe^3+^–XO complexes that were generated using known quantities of FeCl_3_ (Table 3).

**Table 3:** Molar absorption coefficients of the ferric ion–xylenol orange complex generated by different hydroperoxides and the amount of Fe^3+^ ions generated per mole of hydroperoxide

Hydroperoxide species	Molar absorption coefficient Fe^3+^–XO complex (M^−1^ cm^−1^)	Fe^3+^/hydroperoxide ratio^*^			
		This study	(Vega et al. 2005)	(Gay and Gebicki 2002)	(Gay et al. 1999a)
CuHP	21,823 ± 238	5.8	5.6	5.8	5.4
HP	9,632 ± 240	2.5		2.9	2.4
13-HPODE	17,711 ± 165	4.7	8.6		2.0
13-HPOTE	17,068 ± 90	4.5	4.6		
15-HPETE	15,838 ± 93	4.2	4.1		
12-HPEPE	11,060 ± 186	2.9			
17-HPDHE	12,307 ± 138	3.3			

^*^The ratio of Fe^3+^/hydroperoxide is defined as the number of Fe^3+^ ions generated by each hydroperoxide species. It was calculated from six experiments by dividing the molar absorption coefficient of the Fe^3+^–XO complex generated by each hydroperoxide species by the molar absorption coefficient of the complex generated by ferric ions (from FeCl_3_) in the same reagent (Gay et al. 1999b, 1999a). Please note that the Fe^3+^/hydroperoxide ratio values obtained from the references were determined using different compositions of the FOX reagent. The molar absorption coefficient of each hydroperoxide represents mean ± SD (*n* = 6)

### Comparison of the FOX assay with conjugated diene method

**Fig. 6:**
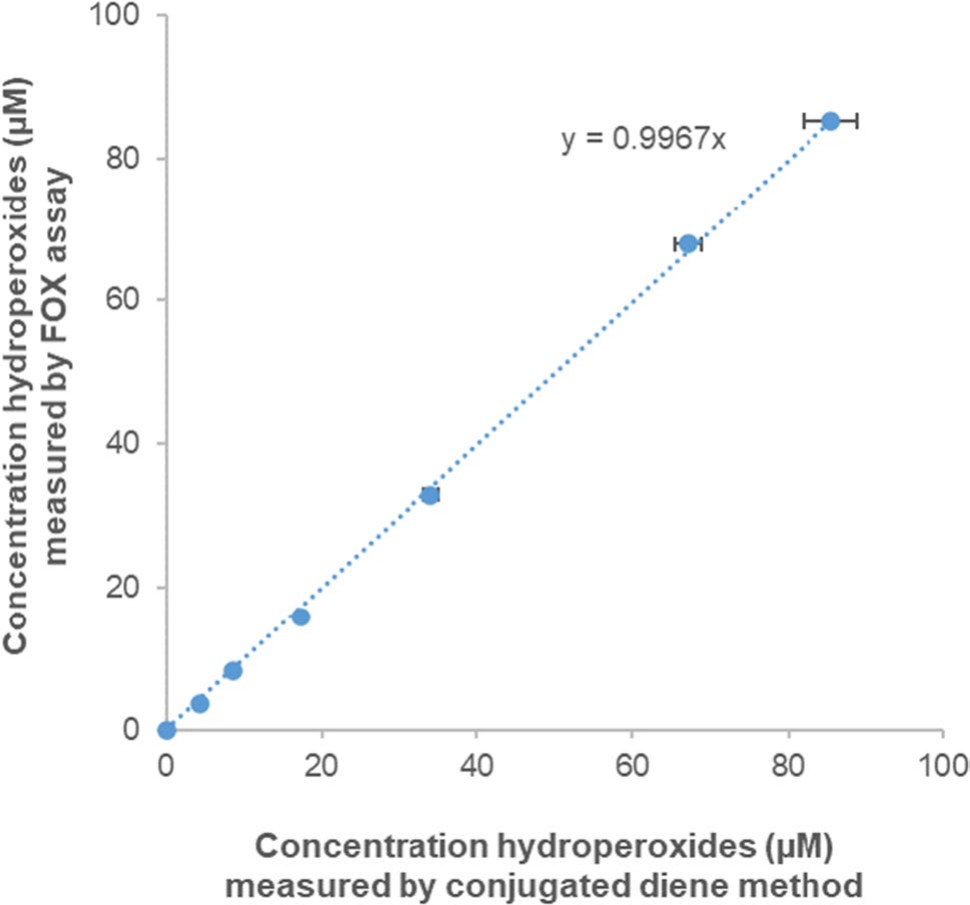
Correlation between the measurement of hydroperoxides produced by conversion of linoleic acid by *B. thailandensis* LOX using the FOX assay and the conjugated diene method. Error bars represent mean ± SD (*n* = 3). When not visible, the error bars are hidden below the markers. Exact concentrations and standard deviations can be found in Table S2

To validate the accuracy of the modified FOX assay and its applicability to measure the activity of LOX enzymes, hydroperoxides produced through the enzymatic conversion of linoleic acid by Bt-LOX were measured with the modified FOX assay and the results were compared with those obtained using the well-established method based on measuring the absorbance of the conjugated diene moiety of the formed FAHPs. The enzymatic reaction conditions were set to obtain full or almost full conversion of the substrate, as observed from a plateau of the curve in the conjugated diene measurement at 234 nm (Fig. S4). The end-point measurement of hydroperoxide concentrations using the two different methods was compared (Table S2) and the results are shown in Fig. 6. A high correlation between hydroperoxide concentrations measured by the modified FOX assay and the conjugated diene method was observed, confirming that the modified FOX assay method is an accurate method to measure LOX activity.

## Discussion

In this study, we modified the FOX assay and evaluated its capacity to be used for rapid screening of LOX activity. The limited linear detection range of the FOX assay, as indicated by previous research (Nielsen et al. 2003; Pinto et al. 2007), poses a disadvantage when it comes to the quantification of LOX-produced FAHPs. In order to broaden the linear detection range of hydroperoxides, the perchloric acid concentration in the FOX reagent was increased compared to the previously reported optimal concentration of 110 mM (Gay and Gebicki 2002). When higher concentrations of perchloric acid are used, ferrous iron becomes less susceptible to oxidation and xylenol orange becomes fully protonated which will slow down the complexation reaction. To reach a similar level of absorbance, higher concentrations of hydroperoxides are needed, which consequently leads to the expansion of the linear detection range. To strike a balance between expanding the linear detection range of hydroperoxides and ensuring adequate assay sensitivity, optimization of the perchloric acid concentration in the FOX reagent was performed. The modification of the FOX assay, by increasing the perchloric acid concentration to 440 mM, has successfully expanded the linear range of hydroperoxide quantification up to fivefold compared to the previously established FOX assay. Importantly, this modification has maintained the molar absorption coefficient of the Fe^3+^–XO complex at a reasonable level, ensuring reliable and precise quantification of hydroperoxides. For instance, the modified FOX assay has demonstrated the ability to quantify down to 4.4 µM of linoleic acid hydroperoxide, as indicated in Table S2. If extremely sensitive detection is required, it is advisable to use 110 mM perchloric acid in the xylenol orange reagent.

To evaluate the applicability of the modified FOX assay for the pH optimum screening of LOXs, the method was applied to CuHP standard samples prepared in different buffers covering a wide pH range. Selection of the buffer used for the enzymatic reaction monitored using the FOX assay is important. Besides not inhibiting the enzyme, the buffer should not contain any compounds that inhibit ferrous oxidation and Fe^3+^ − XO complexation. Compounds containing phosphate groups, for example, should be avoided, as they can bind either to the ferrous ion or the ferric ion to form iron salts, thus inhibiting ferrous oxidation and the formation of Fe^3+^ − XO complex (Tadolini and Sechi 1987; Wang et al. 2021). The modified FOX assay demonstrated applicability across a wide pH range, from pH 3 to 10. Nevertheless, the sensitivity of the assay depends on the type of sample buffer used and its pH. Lowering the sample pH within the same buffer type resulted in a decrease in the molar absorption coefficient of the Fe^3+^ − XO complex. In the case of Tris–HCl buffer from pH 10 to 8 and of Bis–Tris buffer from pH 7 to 6, for example, the reduction in molar absorption coefficient may be attributed to the use of hydrochloric acid for pH adjustment during buffer preparation. This is because chloride ions can coordinate with both ferrous and ferric ions in organic solvents (in this case in methanol/water 9:1) to form ferrous/ferric-chloride complexes (Inoue et al. 2022), thereby inhibiting the oxidation of ferrous iron and the formation of the Fe^3+^ − XO complex. Another possible explanation is that sample buffers with different pH values, when added to the assay reagent, may slightly affect the final pH of the assay mixture. It has indeed been reported that the color development in the FOX assay is influenced by the pH of the reaction mixture. For instance, when using 25 mM sulfuric acid in the xylenol orange reagent, the optimal pH for maximum color development was found to be 1.7–1.8 (Banerjee et al. 2003). When using perchloric acid at 110 mM in the xylenol orange reagent, the pH of the reaction mixture is 1.1 (Gay and Gebicki 2002). In this study, when using 440 mM perchloric acid in the xylenol orange reagent, the final pH of the reaction mixtures ranged from 0.50 to 0.60, depending on the buffer type and its pH (Fig. S5). This suggests that the pH of the reaction mixture may not fall within the optimal range for color development. In addition, evaluating the effect of the final pH of the mixture on color development is challenging, as different types of buffers yielded different responses (Fig. S5). Overall, the modified FOX assay has demonstrated its capability to measure hydroperoxides across a wide range of sample pH; it is, however, important to use a standard curve prepared in the same buffer as used in the sample due to the sensitivity of the assay to the type of sample buffer and its pH.

The reliability of measuring FAHPs produced by LOX using the previously established FOX assay is limited due to the varying reactivity of different FAHP species (such as linoleic, linolenic, and arachidonic hydroperoxides) compared to the commonly used standard compounds, like CuHP and HP (Gay et al. 1999a; Vega et al. 2005). Notably, the response of other FAHP species has not been reported yet. To investigate the sensitivity of the modified FOX assay toward different hydroperoxide species, various concentrations of FAHPs, CuHP, and HP have been measured. The results showed that the modified FOX assay is capable of measuring various hydroperoxide species, thus, the method can be used effectively for screening substrate preference of LOXs. Nevertheless, different hydroperoxide species exhibit different reactivity in the modified FOX assay due to the different number of ferric ions generated by each –OOH group. The number of ferric ions generated by each –OOH group from different hydroperoxide species can be explained based on the reaction pathways of hydroperoxides in the presence of Fe^2+^. Three categories of hydroperoxides can be distinguished (Gay et al. 1999a). The first class produces approximately 2.5 mol of ferric ions per mole of hydroperoxide, with hydrogen peroxide being the only member reported in this class (Gay et al. 1999a). Hydrogen peroxide oxidizes ferrous ions to ferric ions while generating hydroxy radicals. The hydroxy radical (HO•) then reacts with xylenol orange to form xylenol orange-hydroxy radical (HOXO•), which subsequently oxidizes ferrous ions to ferric ions (Gay et al. 1999a). In addition, the HO• can directly oxidize ferrous ions to ferric ions. However, the previous proposed mechanism (Gay et al. 1999a) does not adequately explain the generation of 2.5 mol of ferric ions per mole of hydrogen peroxide. We suggest that other reactions (e.g., via electron transfer from HO• to form XO•, followed by O_2_ coupling to the formed XO•) contribute to the generation of > 2 of ferric ions per hydrogen peroxide molecule. However, the exact mechanism remains to be studied. The second class produces approximately 2.0 mol of ferric ions per mole of hydroperoxide, as previously reported for bovine serum albumin hydroperoxide (Gay et al. 1999a). In this class, each hydroperoxide molecule first oxidizes one ferrous ion to a ferric ion, generating an alkoxyl radical (RO•). The RO• then oxidizes another ferrous ion to a ferric ion, yielding an alcohol (ROH). The third class produces more than 2.5 mol of hydroperoxide, but the underlying mechanisms of these reactions have not been revealed yet. This class is represented, for example, by CuHP and *t*-butyl hydroperoxide (Gay et al. 1999a).

Based on our results, HP yielded 2.5 ferric irons (Table 3), which is in agreement with a previous report that found values between 2.2 and 2.7 depending on the acid used in the xylenol orange reagent (Gay et al. 1999a), and CuHP generated 5.8 ferric irons per mole of hydroperoxide (Table 3); this is in accordance with some previous reports that classified CuHP as belonging to the third class of hydroperoxides (Gay et al. 1999a; Gay and Gebicki 2002; Vega et al. 2005). Finally, the various FAHPs tested, which differed in carbon chain length and amount of double bonds, generated different amounts of ferric ions. 13-HPODE, 13-HPOTE, and 15-HPETE generated 4.2–4.7 ferric ions per mole of hydroperoxide, while 12-HPEPE and 17-HPDHE produced respectively 2.9 and 3.3 ferric ions per mole of hydroperoxide. These results indicated that all tested FAHPs belong to the third class of hydroperoxides. Variation in the number of ferric ions generated by each —OOH group in FAHP was also observed across different studies for 13-HPODE. However, the reason for this variation remains unclear.

**Fig. 7:**
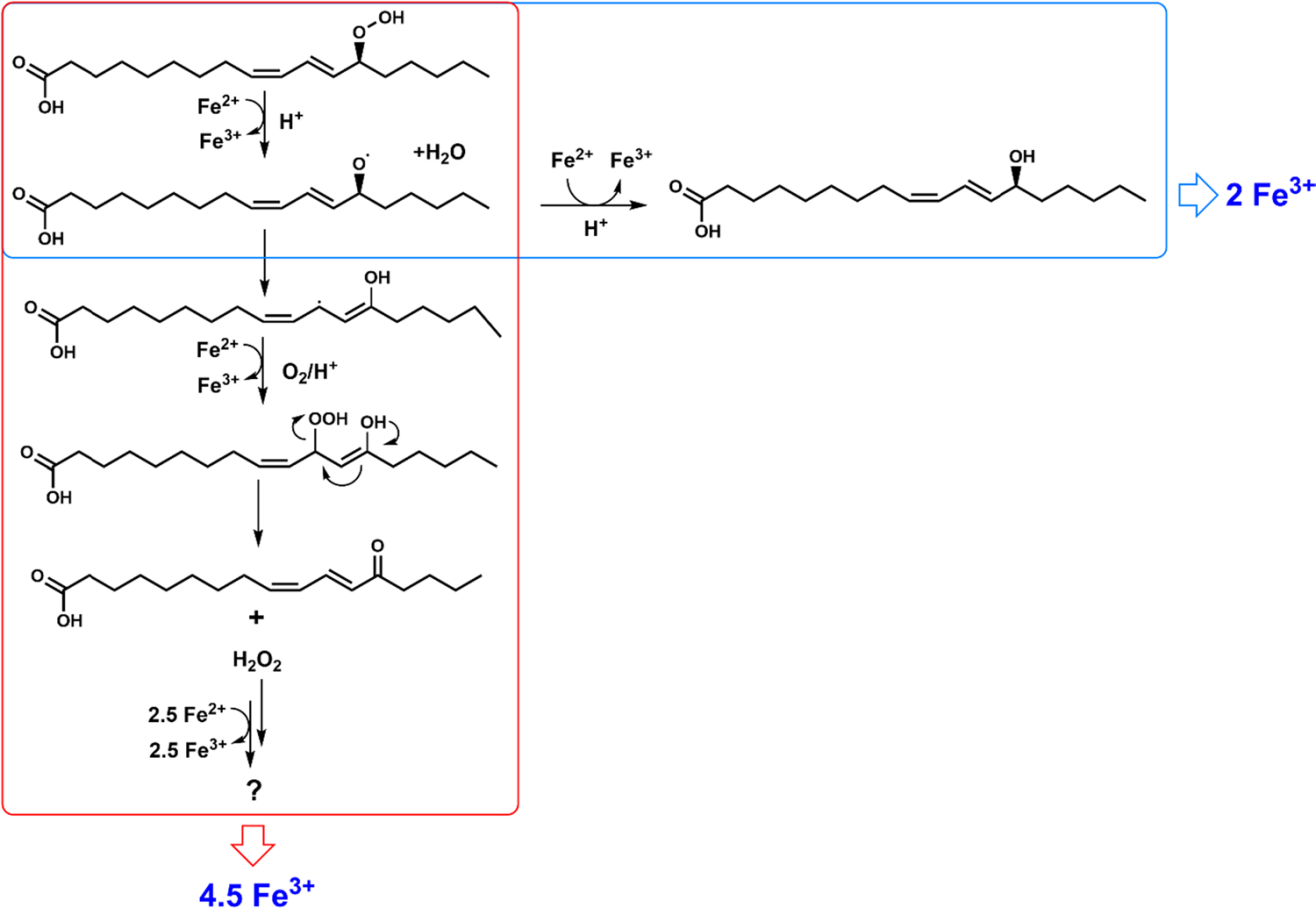
Proposed mechanisms for the generation of 2.0 or 4.5 mol of ferric ions in the FOX assay from 1 mol of 13-HPODE. Generation of 2 mol of ferric ions is indicated by a blue box. Generation of 4.5 mol of ferric ions is indicated by a red box. This involves the reaction of Fe^2+^ with 13-HPODE, yielding an alcohol radical that can oxidize ferrous ion and yield a stoichiometric amount of hydrogen peroxide (H_2_O_2_). Finally, the H_2_O_2_ can generate 2.5 Fe^3+^ (as demonstrated in this study in Table 3). Please note that the actual reaction mechanism may be more complex, as there are various possibilities for radical rearrangement, as described in Fig. S6, which can lead to different amounts of ferric ions being generated

A possible mechanistic explanation for why all the tested FAHPs produced more than 2.5 ferric ions per mole of hydroperoxide is that the alkoxyl radicals (RO•) formed during the colorimetric assay (Gay et al. 1999a) (Eq. 2) rearrange to form carbon-centered alcohol radicals (•ROH) (Eq. 3), which, in the presence of oxygen and under acidic conditions, can oxidize ferrous ion and yield a stoichiometric amount of hydrogen peroxide (Eq. 4) (Fig. 7). This hydrogen peroxide can then produce 2.5 additional ferrous ions as previously described. It is important to note that the reaction pathway shown in Fig. 7 is a simplification, and in practice, radicals might rearrange to form a more complex collection of products, as described in Fig. S6. The type of radical rearrangements that occur will affect the amounts of ferric ions generated. So, depending on the relative contribution of the individual reaction pathways from each of the FAHPs, theoretically 2.0–6.5 ferric ions can be formed. To fully rationalize the amount of ferric ions generated by the different hydroperoxides, the relative contribution of all different reaction pathways should be evaluated, which is beyond the scope of this study.

2 $${\text{ROOH}}+{{\text{Fe}}}^{2+}+{{\text{H}}}^{+}\to {{\text{Fe}}}^{3+}+{{\text{H}}}_{2}{\text{O}}+{\text{RO}}\cdot$$

3 $${\text{RO}}\cdot \to \cdot {\text{ROH}}$$

4 $$\cdot {\text{ROH}}+{{\text{Fe}}}^{2+}+{{\text{O}}}_{2}+{{\text{H}}}^{+}\to {\text{RO}}+{{\text{Fe}}}^{3+}+{{\text{H}}}_{2}{{\text{O}}}_{2}$$

Overall, the modified FOX assay has demonstrated its ability to measure various hydroperoxide species. However, the sensitivity of the assay toward different hydroperoxide species necessitates calibration using the respective standard compound. When financial constraints hinder the use of FAHPs standard compounds during routine substrate preference screening for LOX activity, the use of a correction factor can be considered. To do so, determination of the molar absorption coefficient of the appropriate standard FAHP under the applied conditions should initially be performed together with another standard (e.g., CuHP or HP) using the same FOX reagent. Following this, CuHP or HP can be used as the standard for routine analysis for measuring LOX activity, with the FAHP concentration being calculated using a correction factor based on its molar absorption coefficient. An example of the application of correction factors in determining the concentration of FAHPs is given in Table S3. The use of a correction factor is quite common in spectrophotometric assays, such as the Bradford assay for protein determination (Sachett et al. 2020; Rekowski et al. 2021).

In conclusion, increasing the perchloric acid concentration to 440 mM in the FOX assay has successfully expanded the linear range of hydroperoxide quantification up to fivefold compared to the previously established method. The modified FOX assay demonstrated applicability across a wide pH range, from pH 3 to 10, and is capable of measuring various hydroperoxide species. Thus, the method can be used effectively for screening the optimum pH and substrate preference of LOXs. To enhance quantification accuracy, it is necessary to create calibration curves using appropriate standard compounds diluted in the same buffer as used for the samples. The strong correlation between hydroperoxide measurements obtained using the modified FOX assay and the commonly used conjugated diene method further confirms the accuracy and robustness of this modified assay. By providing a high-throughput and reliable screening method, this modified FOX assay could facilitate the screening of LOX activity in order to exploit their potential as industrial biocatalysts.

## Supplementary Information

Below is the link to the electronic supplementary material.

Supplementary file1 (PDF 624 KB)

## Data Availability

The datasets generated and analyzed in the current study are available from the corresponding author on request.

## References

[CR1] An JU, Kim BJ, Hong SH, Oh DK (2015) Characterization of an omega-6 linoleate lipoxygenase from *Burkholderia**thailandensis* and its application in the production of 13-hydroxyoctadecadienoic acid. Appl Microbiol Biotechnol 99:5487–5497. 10.1007/s00253-014-6353-8 25586578 10.1007/s00253-014-6353-8

[CR2] An JU, Hong SH, Oh DK (2018) Regiospecificity of a novel bacterial lipoxygenase from *Myxococcus**xanthus* for polyunsaturated fatty acids. Biochim Biophys Acta Mol Cell Biol Lipids 1863:823–833. 10.1016/j.bbalip.2018.04.014 29684557 10.1016/j.bbalip.2018.04.014

[CR3] Anthon GE, Barrett DM (2001) Colorimetric method for the determination of lipoxygenase activity. J Agric Food Chem 49:32–37. 10.1021/jf000871s 11170556 10.1021/jf000871s

[CR4] Axelrod B, Cheesbrough TM, Laakso S (1981) Lipoxygenase from soybeans: EC 1.13.11.12 linoleate: oxygen oxidoreductase. Methods Enzymol 71:441–451. 10.1016/0076-6879(81)71055-3

[CR5] Banerjee D, Madhusoodanan UK, Sharanabasappa M, Ghosh S, Jacob J (2003) Measurement of plasma hydroperoxide concentration by FOX-1 assay in conjunction with triphenylphosphine. Clin Chim Acta 337:147–152. 10.1016/J.CCCN.2003.08.004 14568191 10.1016/j.cccn.2003.08.004

[CR6] Banthiya S, Kalms J, Galemou Yoga E, Ivanov I, Carpena X, Hamberg M, Kuhn H, Scheerer P (2016) Structural and functional basis of phospholipid oxygenase activity of bacterial lipoxygenase from *Pseudomonas**aeruginosa*. Biochim Biophys Acta Mol Cell Biol Lipids 1861:1681–1692. 10.1016/j.bbalip.2016.08.002 10.1016/j.bbalip.2016.08.002 27500637

[CR7] Belleza OJV, Villaraza AJL (2014) Ion charge density governs selectivity in the formation of metal–xylenol orange (M–XO) complexes. Inorg Chem Commun 47:87–92. 10.1016/J.INOCHE.2014.07.024

[CR8] Berkeley HD, Galliard T (1976) Measurement of lipoxygenase activity in crude and partially purified potato extracts. Phytochemistry 15:1475–1479. 10.1016/S0031-9422(00)88919-0

[CR9] Bou R, Codony R, Tres A, Decker EA, Guardiola F (2008) Determination of hydroperoxides in foods and biological samples by the ferrous oxidation-xylenol orange method: a review of the factors that influence the method’s performance. Anal Biochem 377:1–15. 10.1016/j.ab.2008.02.029 18358821 10.1016/j.ab.2008.02.029

[CR10] Browne RW, Armstrong D (1998) Separation of hydroxy and hydroperoxy polyunsaturated fatty acids by high-pressure liquid chromatography. Methods Mol Biol 108:147–155. 10.1385/0-89603-472-0:147 9921525 10.1385/0-89603-472-0:147

[CR11] Cho YS, Kim HS, Kim CH, Cheon HG (2006) Application of the ferrous oxidation-xylenol orange assay for the screening of 5-lipoxygenase inhibitors. Anal Biochem 351:62–68. 10.1016/j.ab.2005.12.025 16442488 10.1016/j.ab.2005.12.025

[CR12] Chrisnasari R, Hennebelle M, Vincken J, Van BWJH, Ewing TA (2022) Bacterial lipoxygenases: biochemical characteristics, molecular structure and potential applications. Biotechnol Adv 61:108046. 10.1016/j.biotechadv.2022.108046 36202263 10.1016/j.biotechadv.2022.108046

[CR13] Corongiu FP, Milia A (1983) An improved and simple method for determining diene conjugation in autoxidized polyunsaturated fatty acids. Chem Biol Interact 44:289–297. 10.1016/0009-2797(83)90056-X 6872094 10.1016/0009-2797(83)90056-x

[CR14] Egmond MR, Vliegenthart JFG, Boldingh J (1972) Stereospecificity of the hydrogen abstraction at carbon atom n-8 in the oxygenation of linoleic acid by lipoxygenases from corn germs and soya beans. Biochem Biophys Res Commun 48:1055–1060. 10.1016/0006-291X(72)90815-7 5066282 10.1016/0006-291x(72)90815-7

[CR15] Fukuzawa K, Nano M, Akai K, Morishige J, Tokumura A, Jisaka M (2009) Measurement of lipid hydroperoxides by the ferric-xylenol orange method (2): application to lipoxygenase assay. J Nutr Sci Vitaminol (tokyo) 55:92–98. 10.3177/jnsv.55.92 19352069 10.3177/jnsv.55.92

[CR16] Gao Y, Zhang Y (2018) Formation and photochemical properties of aqueous brown carbon through glyoxal reactions with glycine. RCS Adv 8:38566–38573. 10.1039/c8ra06913a 10.1039/c8ra06913a PMC9090559 35559051

[CR17] Gay CA, Gebicki JM (2002) Perchloric acid enhances sensitivity and reproducibility of the ferric–xylenol orange peroxide assay. Anal Biochem 304:42–46. 10.1006/ABIO.2001.5566 11969187 10.1006/abio.2001.5566

[CR18] Gay C, Collins J, Gebicki JM (1999a) Hydroperoxide assay with the ferric-xylenol orange complex. Anal Biochem 273:149–155. 10.1006/ABIO.1999.4208 10469484 10.1006/abio.1999.4208

[CR19] Gay C, Collins J, Gebicki JM (1999b) Determination of iron in solutions with the ferric-xylenol orange complex. Anal Biochem 273:143–148. 10.1006/ABIO.1999.4207 10469483 10.1006/abio.1999.4207

[CR20] Gigot C, Ongena M, Fauconnier ML, Wathelet JP, du Jardin P, Thonart P (2010) The lipoxygenase metabolic pathway in plants: potential for industrial production of natural green leaf volatiles. Biotechnol Agron Soc Environ 14:451–460

[CR21] Goloshchapova K, Stehling S, Heydeck D, Blum M, Kuhn H (2018) Functional characterization of a novel arachidonic acid 12S-lipoxygenase in the halotolerant bacterium *Myxococcus**fulvus* exhibiting complex social living patterns. Microbiol Open 8:1–17. 10.1002/mbo3.775 10.1002/mbo3.775 PMC6612559 30560563

[CR22] Griffiths G, Leverentz M, Silkowski H, Gill N, Sá Nchez-Serrano JJ (2000) Lipid hydroperoxide levels in plant tissues. J Exp Bot 51:1363–1370. 10.1093/jexbot/51.349.1363 10944149

[CR23] Hamberg M, Samuelsson B (1967) On the specificity of the oxygenation of unsaturated fatty acids catalyzed by soybean lipoxidase. J Biol Chem 242:5329–5335. 10.1016/S0021-9258(18)99432-9 6070850

[CR24] Hamberg M, Su C, Oliw E (1998) Manganese lipoxygenase. Discovery of a bis-allylic hydroperoxide as product and intermediate in a lipoxygenase reaction. J Biol Chem 273:13080–13088. 10.1074/JBC.273.21.13080 9582346 10.1074/jbc.273.21.13080

[CR25] Hermes-Lima M, Willmore WG, Storey KB (1995) Quantification of lipid peroxidation in tissue extracts based on Fe(III)xylenol orange complex formation. Free Radic Biol Med 19:271–280. 10.1016/0891-5849(95)00020-X 7557541 10.1016/0891-5849(95)00020-x

[CR26] Hu J, Jin Z, Chen TY, Polley JD, Cunningham MF, Gross RA (2014) Anionic polymerizable surfactants from biobased ω-hydroxy fatty acids. Macromolecules 47:113–120. 10.1021/MA401292C

[CR27] Inoue D, Komatsu T, Niwa H, Ina T, Nitani H, Abe H, Moritomo Y (2022) Coordination states of Fe^3+^ and Fe^2+^ dissolved in organic solvents. J Physical Soc Japan 91. 10.7566/JPSJ.91.094605

[CR28] Jiang ZY, Hunt JV, Wolff SP (1992) Ferrous ion oxidation in the presence of xylenol orange for detection of lipid hydroperoxide in low density lipoprotein. Anal Biochem 202:384–389. 10.1016/0003-2697(92)90122-N 1519766 10.1016/0003-2697(92)90122-n

[CR29] Kim SE, Lee J, An JU, Kim TH, Oh CW, Ko YJ, Krishnan M, Choi J, Yoon DY, Kim Y, Oh DK (2022) Regioselectivity of an arachidonate 9*S*-lipoxygenase from *Sphingopyxis macrogoltabida* that biosynthesizes 9*S*,15*S*- and 11*S*,17*S*-dihydroxy fatty acids from C20 and C22 polyunsaturated fatty acids. Biochim Biophys Acta (BBA) - Mol Cell Biol Lipids 1867:159091. 10.1016/J.BBALIP.2021.159091 10.1016/j.bbalip.2021.159091 34902567

[CR30] Kim C, Eom JB, Jung S, Ji T (2016) Detection of organic compounds in water by an optical absorbance method. Sensors (Basel) 16. 10.3390/S16010061 10.3390/s16010061 PMC4732094 26742043

[CR31] Krukowski S, Karasiewicz M, Kolodziejski W (2017) Convenient UV-spectrophotometric determination of citrates in aqueous solutions with applications in the pharmaceutical analysis of oral electrolyte formulations. J Food Drug Anal 25:717–722. 10.1016/J.JFDA.2017.01.009 28911657 10.1016/j.jfda.2017.01.009 PMC9328833

[CR32] Kuhn H, Gotze R, Schewe T, Rapoport SM (1981) Quasi-lipoxygenase activity of haemoglobin: a model for liproxygenases. Eur J Biochem 120:161–168. 10.1111/j.1432-1033.1981.tb05684.x 6796416 10.1111/j.1432-1033.1981.tb05684.x

[CR33] Le Guern F, Mussard V, Gaucher A, Rottman M, Prim D (2020) Fluorescein derivatives as fluorescent probes for pH monitoring along recent biological applications. Int J Mol Sci 21:1–23. 10.3390/ijms21239217 10.3390/ijms21239217 PMC7729466 33287208

[CR34] Lehnert N, Solomon EI (2003) Density-functional investigation on the mechanism of H-atom abstraction by lipoxygenase. J Biol Inorg Chem 8:294–305. 10.1007/S00775-002-0415-6 12589565 10.1007/s00775-002-0415-6

[CR35] Li Y, Schwarz PB (2018) Use of a ferrous oxidation-xylenol orange (FOX) assay to determine lipoxygenase activity in barley and malt. https://doi-org.ezproxy.library.wur.nl/101094/ASBCJ-2012-1011-01 70:287–289. 10.1094/ASBCJ-2012-1011-01

[CR36] Liosi GM, Dondi D, Vander Griend DA, Lazzaroni S, D’Agostino G, Mariani M (2017) Fricke-gel dosimeter: overview of xylenol orange chemical behavior. Radiat Phys Chem 140:74–77. 10.1016/j.radphyschem.2017.01.012

[CR37] Liu C, Liu F, Cai J, Xie UW, Long TE, Turner SR, Lyons A, Gross RA (2012) Polymers from fatty acids: poly(co-hydroxyl tetradecanoic acid) synthesis and physico-mechanical studies. ACS Symp Ser 1105:131–150. 10.1021/BK-2012-1105.CH009 10.1021/bm2007554 21793591

[CR38] Lu J, Zhang C, Leong HY, Show PL, Lu F, Lu Z (2020) Overproduction of lipoxygenase from *Pseudomonas**aeruginosa* in *Escherichia**coli* by auto-induction expression and its application in triphenylmethane dyes degradation. J Biosci Bioeng 129:327–332. 10.1016/j.jbiosc.2019.09.006 31585857 10.1016/j.jbiosc.2019.09.006

[CR39] Musakhanian J, Rodier JD, Dave M (2022) Oxidative stability in lipid formulations: a review of the mechanisms, drivers, and inhibitors of oxidation. AAPS PharmSciTech 23. 10.1208/s12249-022-02282-0 10.1208/s12249-022-02282-0 35596043

[CR40] Mutlu H, Meier MAR (2010) Castor oil as a renewable resource for the chemical industry. Eur J Lipid Sci Technol 112:10–30. 10.1002/EJLT.200900138

[CR41] Newie J, Andreou A, Neumann P, Einsle O, Feussner I, Ficner R (2016) Crystal structure of a lipoxygenase from *Cyanothece* sp. may reveal novel features for substrate acquisition. J Lipid Res 57:276–286. 10.1194/jlr.M064980 26667668 10.1194/jlr.M064980 PMC4727423

[CR42] Nielsen NS, Timm-Heinrich M, Jacobsen C (2003) Comparison of wet-chemical methods for determination of lipid hydroperoxides. J Food Lipids 10:35–50. 10.1111/J.1745-4522.2003.TB00004.X

[CR43] Pinto MDC, Tejeda A, Duque AL, Macías P (2007) Determination of lipoxygenase activity in plant extracts using a modified ferrous oxidation−xylenol orange assay. J Agric Food Chem 55:5956–5959. 10.1021/jf070537x 17602650 10.1021/jf070537x

[CR44] Qi Y-K, Zheng Y-C, Zhang Z-J, Xu J-H (2020) Efficient transformation of linoleic acid into 13(*S*)-hydroxy-9,11-(*Z, *E)-octadecadienoic acid using putative lipoxygenases from Cyanobacteria. ACS Sustain Chem Eng 8:5558–5565. 10.1021/acssuschemeng.9b07457

[CR45] Qian H, Xia B, He Y, Lu Z, Bie X, Zhao H, Zhang C, Lu F (2017) Expression, purification, and characterization of a novel acidic lipoxygenase from *Myxococcus**xanthus*. Protein Expr Purif 138:13–17. 10.1016/j.pep.2017.05.006 28552618 10.1016/j.pep.2017.05.006

[CR46] Rekowski A, Langenkämper G, Dier M, Wimmer MA, Scherf KA, Zörb C (2021) Determination of soluble wheat protein fractions using the Bradford assay. Cereal Chem 98:1059–1065. 10.1002/cche.10447

[CR47] Romero MV, Barrett DM (1997) Rapid methods for lipoxygenase assay in sweet corn. J Food Sci 62:696–700. 10.1111/j.1365-2621.1997.tb15438.x

[CR48] Sachett A, Gallas-Lopes M, Conterato GMM, Benvenutti R, Herrmann AP, Piato A (2020) Quantification of thiobarbituric acid reactive species (TBARS) optimized for zebrafish brain tissue fish behavior and physiology LAPCOM. protocols. 10.17504/protocols.io.bjp8kmrw

[CR49] Scotti M, Arosio P, Brambilla E, Gallo S, Lenardi C, Locarno S, Orsini F, Pignoli E, Pedicone L, Veronese I (2022) How xylenol orange and ferrous ammonium sulphate influence the dosimetric properties of PVA–GTA Fricke gel dosimeters: a spectrophotometric study. Gels 8. 10.3390/gels8040204 10.3390/gels8040204 PMC9025870 35448105

[CR50] Song JW, Jeon EY, Song DH, Jang HY, Bornscheuer UT, Oh DK, Park JB (2013) Multistep enzymatic synthesis of long-chain α, ω-dicarboxylic and ω-hydroxycarboxylic acids from renewable fatty acids and plant oils. Angew Chem Int Ed 52:2534–2537. 10.1002/anie.201209187 10.1002/anie.201209187 23362232

[CR51] Straub KL, Benz M, Schink B (2001) Iron metabolism in anoxic environments at near neutral pH. FEMS Microbiol Ecol 34:181–186. 10.1111/j.1574-6941.2001.tb00768.x 11137597 10.1111/j.1574-6941.2001.tb00768.x

[CR52] Šuňovská A, Horník M, Marešová J, Pipíška M, Augustín J (2012) 137Cs uptake and translocation in leafy vegetable: a study with *Lactuca**sativa* L. grown under hydroponic conditions. Nova Biotechnol Et Chim 11:153–166. 10.2478/V10296-012-0018-8

[CR53] Tadolini B, Sechi AM (1987) Iron oxidation in Mops buffer. Effect of phosphorus containing compounds. Free Radic Res Commun 4:161–172. 10.3109/10715768709088101 3148494 10.3109/10715768709088101

[CR54] Timabud T, Sanitchon J, Pongdontri P (2013) A modified ferrous oxidation-xylenol orange assay for lipoxygenase activity in rice grains. Food Chem 141:2405–2411. 10.1016/J.FOODCHEM.2013.05.037 23870974 10.1016/j.foodchem.2013.05.037

[CR55] Vega M, Karbounea S, Husson F, Kermasha S (2005) Optimization of enzymatic assay for the measurement of lipoxygenase activity in organic solvent media. JAOCS 82:817–823. 10.1007/s11746-005-1149-3

[CR56] Wang Q, Liao Z, Yao D, Yang Z, Wu Y, Tang C (2021) Phosphorus immobilization in water and sediment using iron-based materials: a review. Sci Total Environ 767. 10.1016/j.scitotenv.2020.144246 10.1016/j.scitotenv.2020.144246 33434847

[CR57] Waslidge NB, Hayes DJ (1995) A colorimetric method for the determination of lipoxygenase activity suitable for use in a high throughput assay format. Anal Biochem 231:354–358. 10.1006/abio.1995.0063 8594985 10.1006/abio.1995.0063

[CR58] Whent M, Ping T, Kenworthy W, Yu L (2010) High-throughput assay for detection of soybean lipoxygenase-1. J Agric Food Chem 58:12602–12607. 10.1021/JF1028784 21121611 10.1021/jf1028784

[CR59] Wolff SP (1994) Ferrous ion oxidation in presence of ferric ion indicator xylenol orange for measurement of hydroperoxides. Methods Enzymol 233:182–189. 10.1016/S0076-6879(94)33021-2

[CR60] Yoshino T, Murakami S, Ogura K (1979) Equilibria of iron(III) complexes with xylenol orange and methylthymol blue. J Inorg Nucl Chem 41:1011–1013. 10.1016/0022-1902(79)80078-0

