# Supplementary material for: Versatile ferrous oxidation–xylenol orange assay for high-throughput screening of lipoxygenase activity
Source: Appl Microbiol Biotechnol. 2024 Mar 18;108:266. doi: 10.1007/s00253-024-13095-5 (PMC10948578; doi:10.1007/s00253-024-13095-5)
Supplement: Supplementary file 1 [file 253_2024_13095_MOESM1_ESM.pdf]

**Applied Microbiology and Biotechnology**

# Versatile Ferrous Oxidation-Xylenol Orange Assay for High-throughput Screening of Lipoxygenase Activity

Ruth Chrisnasari[1,2,3], Tom A. Ewing[2*], Roelant Hilgers[1], Willem J. H. van Berkel[1], Jean-Paul Vincken[1] and Marie Hennebelle[1*]

[1] Laboratory of Food Chemistry, Wageningen University & Research, Bornse Weilanden 9, 6708 WG, Wageningen, The Netherlands
[2] Wageningen Food & Biobased Research, Wageningen University & Research, Bornse Weilanden 9, 6708 WG, Wageningen, The Netherlands
[3] Faculty of Biotechnology, University of Surabaya (UBAYA), Surabaya, Indonesia 60293

[*] corresponding author: tom.ewing@wur.nl, marie.hennebelle@wur.nl

## Supplementary

**Table S1** The gene sequence of *B. thailandensis* LOX with codons optimized for expression in *E. coli*

| **Gene sequence*** |
|---|
| CATATGGTGAACCACAAGACCGGTAGCAACATGAACCGTCGTGACCTGATCAAATTCCTGAGCTTTGCGGCGAGCGGT<br>ACCGCGTTTGCGGGTCTGGTTCGTAGCACCCTGAGCAGCCCGGCGGCGAGCAGCATTACCGCGAGCCCGCGTACC<br>CTGGATGCGGGTATCGGCATTAGCAGCCCGCAGGCGGTGCGTGCGGCGGCGCCGGTTCTGCCGCAAAAGGATACC<br>GCGGCGGGTCGTATTGCGCGTGCGGGCTTTCTGGCGACCCAGCGTCTGAGCTACATCTGGACCGAGCATGTGCCG<br>ACCGCGAGCGGTATTCCGCTGGCGCTGGTTACCCCGCAAGATCTGCCGACCATCGAGTGGCTGATTAAGTTCATC<br>GCGATTGTGGTTGGTGTGATCGAAAACTTTCTGGGCAGCGCGCCGGCGACCGCGGTGGCGCTGTGGCGTGACCAG<br>TTCGCGAAAATTCGTGTTGACCTGCTGTCCCTGGAGAACCTGTATAGCGACCTGACCCACGATCCGAACCTGCAA<br>GATCCGGTGGCGATTGCGCAGGCGGCGAGCATCCAAGCGGCGCTGATTGCGCTGCTGGCGAACGTGGGTGTTCTG<br>AGCAAGGACATCATTAGCCGTCTGGGCGAAATCGTTAGCAACCACGATACCCGTAGCGAGGAAAACTTCAAAGCG<br>CTGTTCAGCACCTTTCCGCTGCCGGATATTAGCGCGGCGTACCAGCGTGACGATCACTTTGCGAGCCTGCGTGTG<br>GCGGGTCAAAACCCGGTTCTGATCAAGCGTATTAGCGGCCTGCCGAGCAAATTCCCGCTGACCAACGCGCAGTTT<br>CAGCAAGTGATGGGTCCGGCGGACAACCTGGTTAGCGCGGCGGCGGAGAACCGTCTGTACCTGCTGGACTATGTG<br>GATAACGGTCTGCTGGCGACCAGCCGTGCGGTTGCGAAGCCGCTGACCGGTATCGGCTACAGCTATGCGCCGATT<br>GCGCTGTTTGCGCTGCCGCGTGGTGGCGCGAGCCTGGTGCCGGTTGCGATCCAGTGCGACCAAGATCCGGCGACC<br>AACCCGCTGTTTCTGCCGGCGGATCCGAGCCAGGAGAGCGCGTACTGGGCGTGGCAGATGGCGAAAACCGTGGTT<br>CAATGCGCGGAGGAAAACTATCACGAAATGTTCGTTCATCTGGCGCGTACCCACCTGGTGACCGGTGCGATCTGC<br>GTTGCGACCCATCGTAACCTGGCGAGCACCCACCCGCTGTATGCGCTGCTGATGCCGCACTTTGAGGGCACCCTG<br>TATATTAACGAACTGGCGGCGCTGACCCTGCTGCCGCCGCTGATGTTCATCGACACCCTGTTTGCGGCGCCGATT<br>CAGCAAACCCAGCAAATGGTGGCGAGCGATCGTCTGGCGTTCGACTTTTACGATCACATGCTGCCGAACGACATC<br>GAAATGCGTGGTGTTGGTGCGGCGAACCTGCCGGATTACCCGTATCGTGACGATGGTCTGCTGATCTGGAACGCG<br>ATTGCGGAGTGGGCGAAGGCGTACGTGGACGTTTACTATAAAAGCGACCAGGATGTGGTTGACGATTATGAACTG<br>CGTAGCTGGGCGGCGGATATCATTGCGAACGGCAAGGTGAAAGGCTTCCGTCCGGTTCGTAGCAAGGCGCAGCTG<br>ATCGACGTGCTGACCATGATCATTTTTACCGCGAGCGCGCAGCACGCGGCGGTTAACTTCAGCCAAAGCGATTTT<br>AGCACCTACGCGCCGGCTCTGAGCGCGCTGCTGTCCGCGCCGGCGCCGACCAGCGCGGTGGGCAAGAGCAAAGCG<br>GACTGGCTGAAAATGCTGCCGCCGCTGGTTAGCGGTATTGAGCGTGTTGCGATCTATGAAATTCTGGCGGGTGTG<br>CAGCACAGCGCGCTGGGTCAATACCGTAGCAACGTTTTCCCGTATCGTCCGCTGATCACCGACCCGGCGATTACC<br>GGTAGCAACGGCCCGCTGGAGCACTTTCGTCAGGCGCTGGGTGATGTTGAAAGCCAAATCAACGCGCGTAACAGC<br>ATTCGTAAAACCCCGTACGAATATCTGCTGCCGAGCCGTATCCCGGCGAGCACCAACATTTAAGCTGAGC |

*Blue letters indicating additional nucleotides added to include sites for restriction enzyme digestion (*NdeI* and *BlpI*).

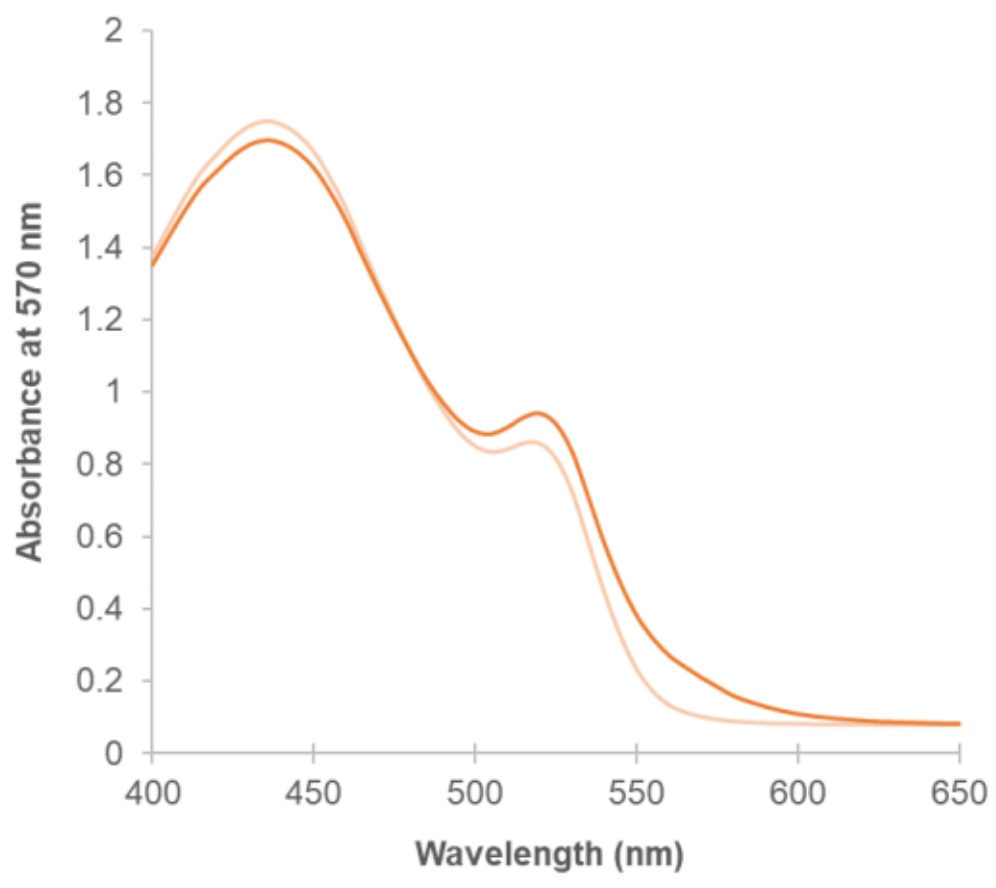


**Fig. S1** Absorption spectra of 0.29 mM xylenol orange tetrasodium salt diluted in 440 mM perchloric acid in methanol/water (9:1). The light orange line represents the xylenol orange reagent without ferrous sulfate, while the dark orange line represents the xylenol orange reagent containing 2.0 mM ferrous sulfate

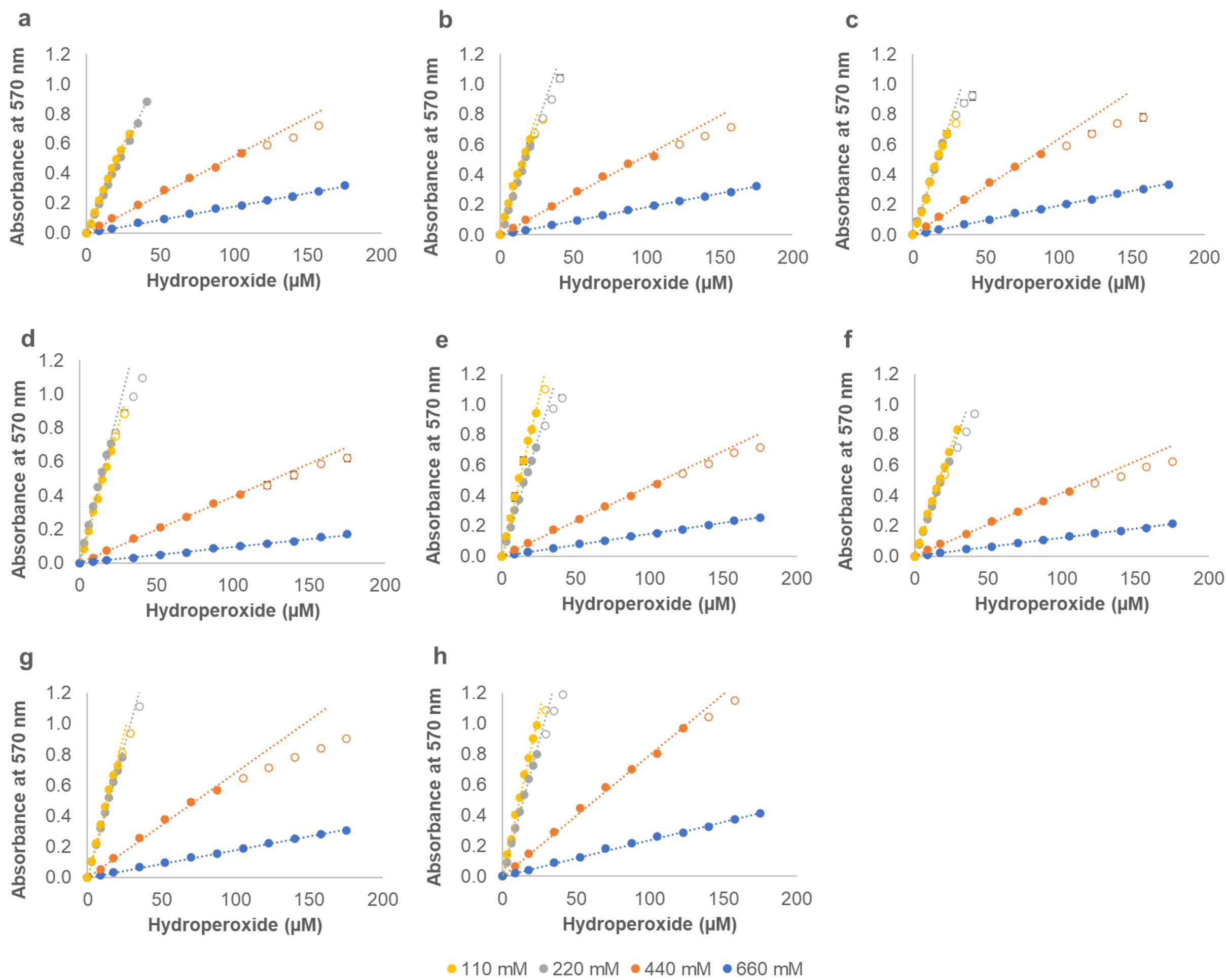


**Fig. S2** The effect of perchloric acid concentration (110, 220, 440, and 660 mM) in the xylenol orange reagent on the detection range of cumene hydroperoxide diluted in different buffers. a) 100 mM citrate pH 3.0, b) 100 mM citrate pH 4.0, c) 100 mM citrate pH 5.0, d) 100 mM Bis-Tris pH 6.0, e) 100 mM Bis-Tris pH 7.0, f) 100 mM Tris-HCl pH 8.0, g) 100 mM Tris-HCl pH 9.0 and h) 100 mM Tris-HCl pH 10.0. The linear ranges are represented by filled circles, while datapoints outside the linear range are indicated by open circles of the same color. Data are presented as the mean ± SD (n = 3). When not visible, the error bars are hidden below the markers

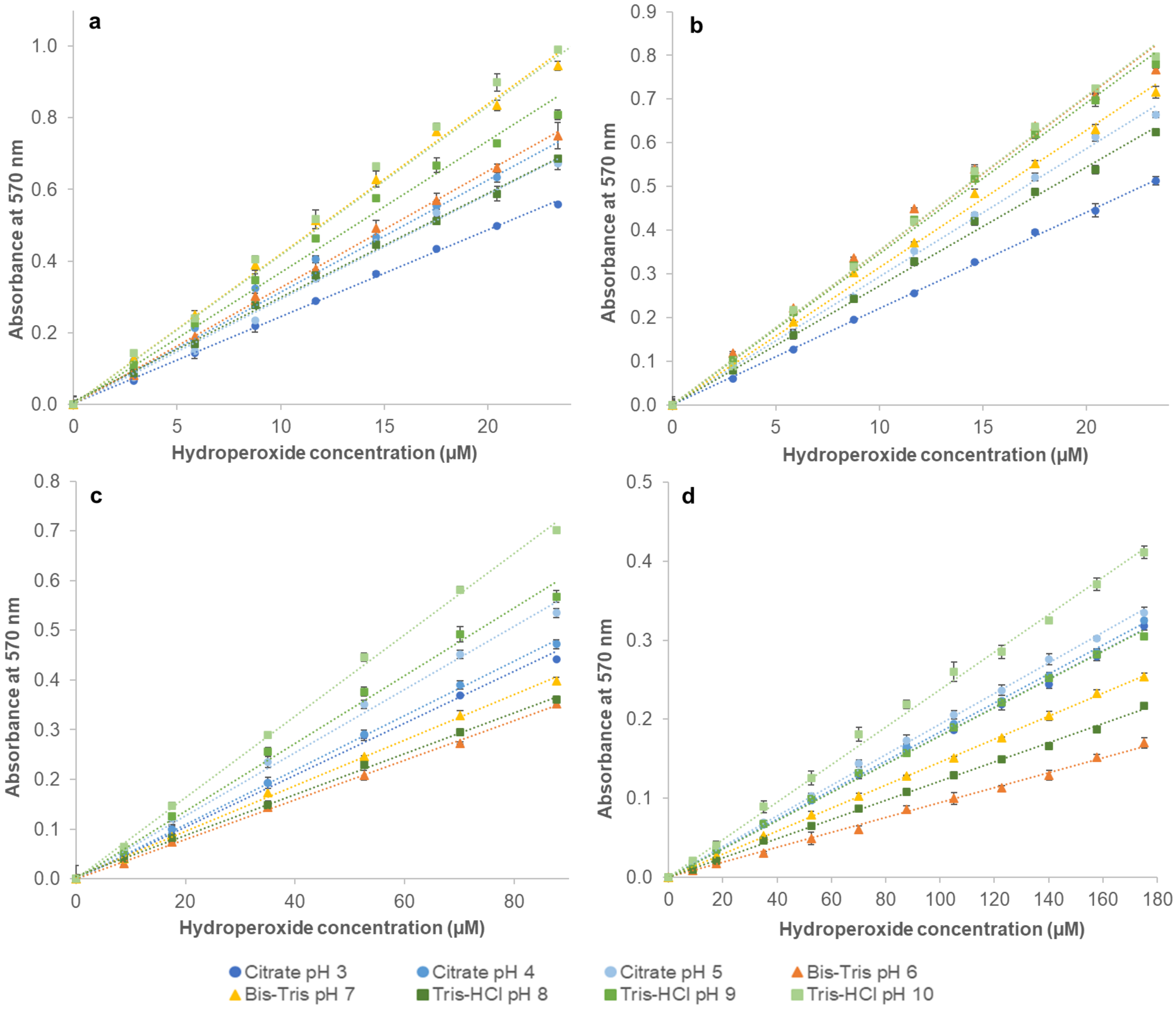


**Fig. S3** The effect of the buffer used to dilute cumene hydroperoxide on the absorbance measured in the FOX assay. FOX reagent was prepared with different perchloric acid concentrations: a) 110 mM, b) 220 mM, c) 440 mM, and d) 660 mM. The buffers used were 100 mM citrate pH 3.0, 4.0, and 5.0, 100 mM Bis Tris-HCl pH 6.0 and 7.0 and 100 mM Tris-HCl pH 8.0, 9.0 and 10.0. Data are presented as the mean ± SD (n = 3)

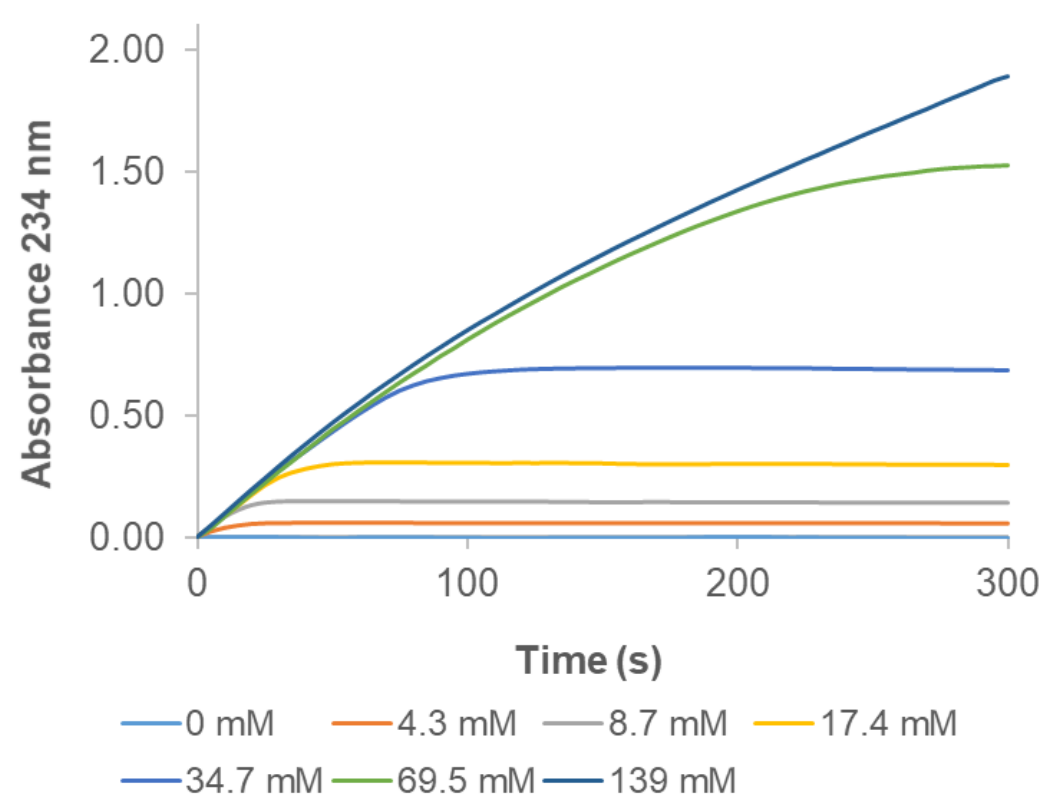


**Figure S4** Time course measurement of LA-derived FAHPs produced by *B. thailandensis* at 234 nm at different concentrations of substrate

**Table S2** Measurement of LA-derived FAHPs produced by *B. thailandensis* LOX using FOX assay and conjugated diene method

| Linoleic Acid substrate (μM) | Fatty acid hydroperoxides (μM) | |
|---|---|---|
| | FOX assay* | Conjugated diene |
| 4.3 | 4.40 ± 0.29 | 3.61 ± 0.47 |
| 8.7 | 8.60 ± 0.24 | 8.34 ± 0.42 |
| 17.4 | 17.36 ± 0.24 | 15.74 ± 0.14 |
| 34.7 | 34.05 ± 0.95 | 32.95 ± 0.11 |
| 69.5 | 67.12 ± 1.78 | 67.86 ± 0.17 |
| 139 | 85.50 ± 3.58 | 85.24 ± 0.52 |

*13-HPODE was used as the standard compound to calculate the amount of FAHPs produced by Bt-LOX. For the conjugated diene method, the concentration of fatty acid hydroperoxide formed was calculated using an extinction coefficient of 25,000 $M^{-1}cm^{-1}$. Data are presented as the mean ± SD (n = 3). Please note that at concentrations of 69.5 μM and 139 μM of linoleic acid, the substrate was not fully converted to FAHPs, as shown in **Fig. S6**.

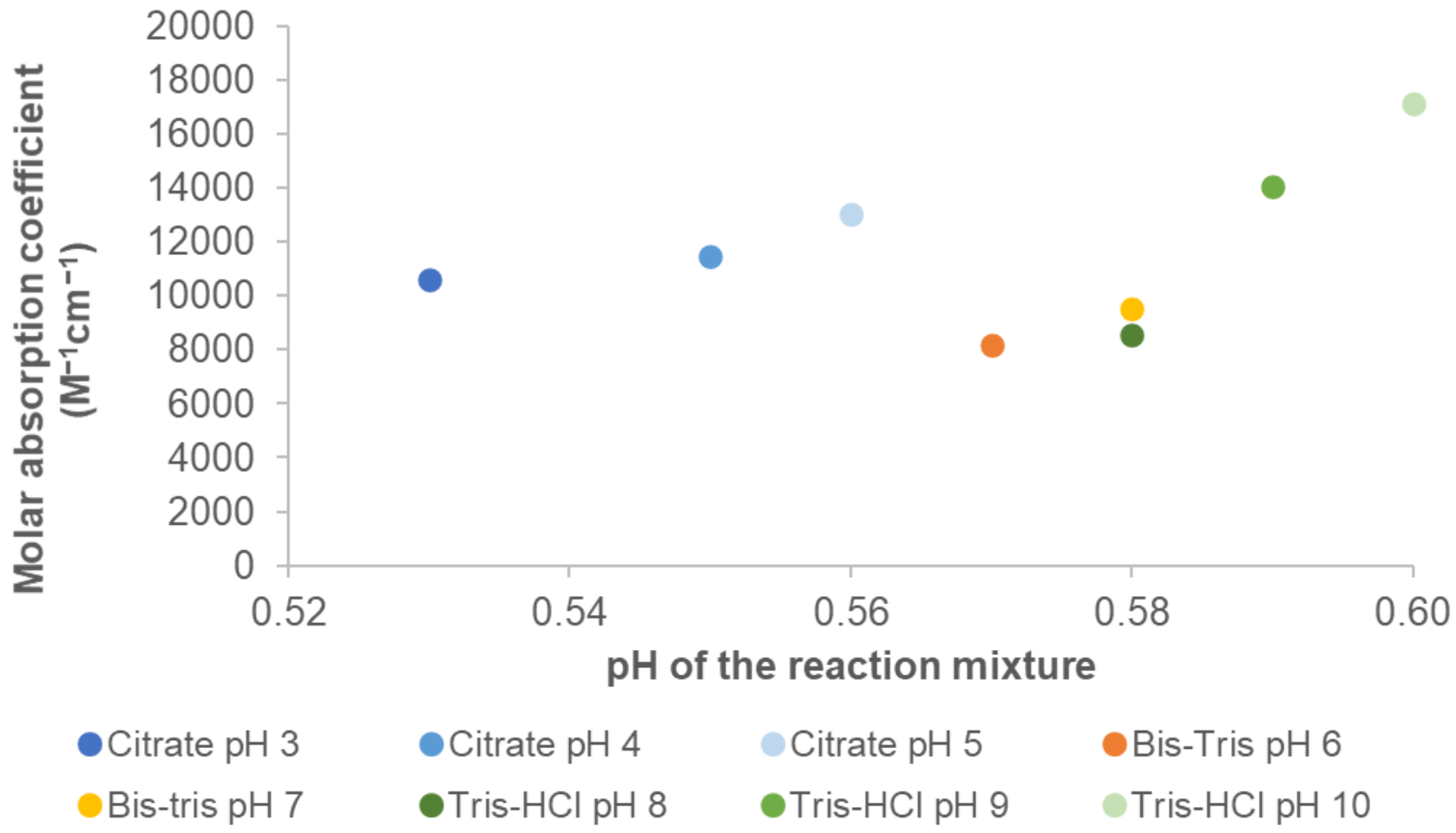


**Fig. S5** The effect of sample buffer and pH on the molar absorption coefficient of ferric-xylenol orange complex measured in FOX reagent containing 440 mM perchloric acid. When not visible, the error bars are hidden below the markers

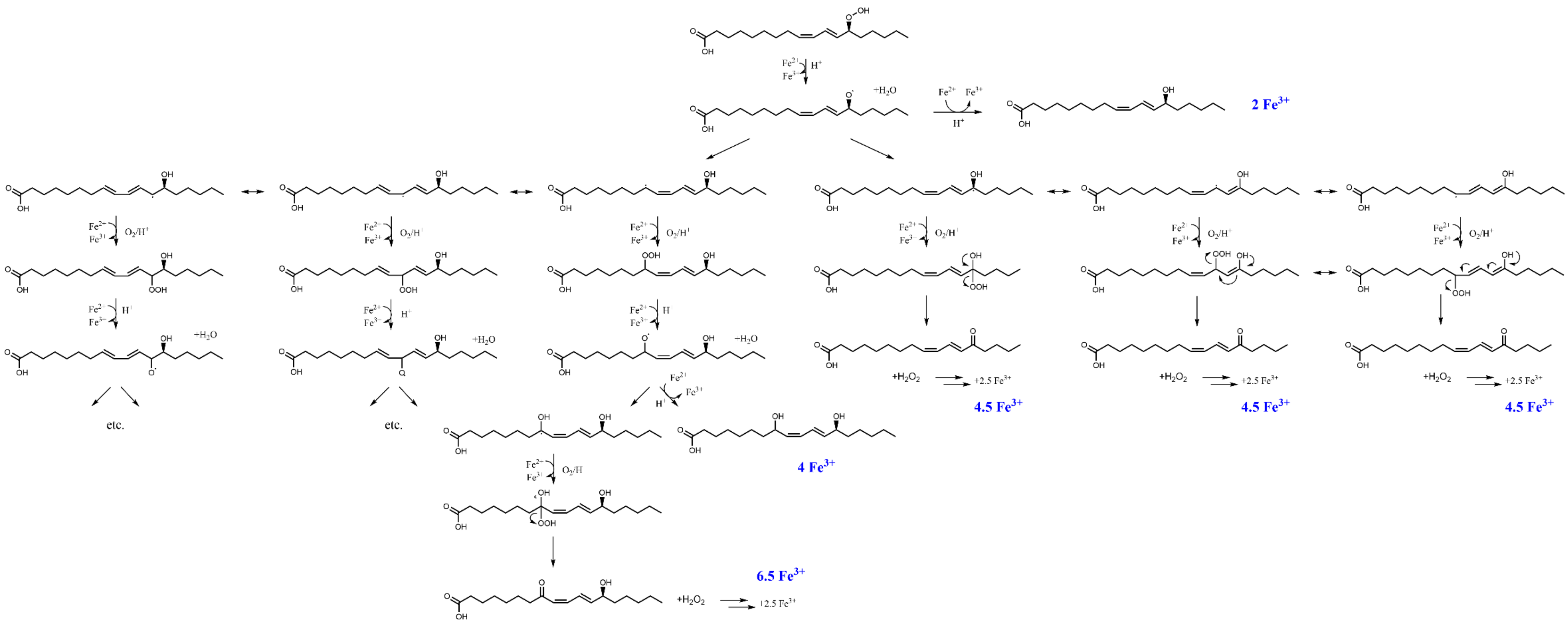


**Fig. S6** Proposed reaction pathways for ferrous iron oxidation by LA-derived FAHPs in the FOX assay. It should be noted that the actual pathways might be even more divergent, further complicating the $Fe^{3+}$/FAHP ratio. Furthermore, many of the theoretically possible pathways will not or only marginally contribute to the overall reaction, and thereby not affect the $Fe^{3+}$/FHAP ratio

**Table S3** The application of a correction factor for determination FAHPs concentration

| Hydroperoxide species | Known concentration (μM) | Absorbance at 570 nm | Correction factor | Concentration (μM) | |
|---|---|---|---|---|---|
| | | | | Calculation using CuHP as standard | With correction factor applied |
| 13-HPODE | 9.08 | 0.0879 ± 0.0017 | 1.2355 | 8.61 ± 0.13 | 10.64 ± 0.16 |
| | 54.51 | 0.5039 ± 0.0065 | | 48.34 ± 0.51 | 59.73 ± 0.63 |
| | 90.84 | 0.7810 ± 0.0060 | | 74.81 ± 0.47 | 92.43 ± 0.58 |
| 13-HPOTE | 9.3 | 0.0978 ± 0.0012 | 1.2916 | 9.56 ± 0.10 | 12.35 ± 0.13 |
| | 55.5 | 0.4751 ± 0.0031 | | 45.59 ± 0.24 | 58.89 ± 0.31 |
| | 92.5 | 0.7635 ± 0.0059 | | 73.14 ± 0.46 | 94.46 ± 0.60 |
| 15-HPETE | 8.3 | 0.0668 ± 0.0019 | 1.3968 | 6.60 ± 0.15 | 9.22 ± 0.22 |
| | 58.1 | 0.4411 ± 0.0051 | | 42.35 ± 0.39 | 59.15 ± 0.55 |
| | 83.1 | 0.6447 ± 0.0029 | | 61.79 ± 0.22 | 86.31 ± 0.31 |
| 12-HPEPE | 14.9 | 0.0984 ± 0.0023 | 1.9635 | 9.61 ± 0.18 | 18.88 ± 0.35 |
| | 59.8 | 0.3407 ± 0.0032 | | 32.76 ± 0.25 | 64.33 ± 0.49 |
| | 89.7 | 0.4829 ± 0.0036 | | 46.34 ± 0.28 | 90.98 ± 0.55 |
| 17-HPDHE | 13.9 | 0.0934 ± 0.0037 | 1.7990 | 9.14 ± 0.28 | 16.44 ± 0.51 |
| | 55.5 | 0.3467 ± 0.0043 | | 33.33 ± 0.34 | 59.96 ± 0.60 |
| | 83.2 | 0.4772 ± 0.0057 | | 45.80 ± 0.45 | 82.38 ± 0.80 |

FAHPs standard compounds at known concentrations were used as samples to assess the applicability of the correction factor in determining concentration of FAHPs. CuHP was employed as calibration sample ($y = 0.0105x - 0.0023$, $R^2 = 0.9981$). The correction factor was calculated by dividing the molar extinction coefficient of CuHP by the molar extinction coefficient of the corresponding standard FAHP, as indicated in Table 3. The real concentration of FAHPs was calculated by multiplying the correction factor with the concentration of FAHPs measured using CuHP as a standard. Data are presented as the mean ± SD (n = 3).
